# Supplementary material for: Timely TGFβ signalling inhibition induces notochord
Source: Nature. 2024 Dec 18;637(8046):673–82. doi: 10.1038/s41586-024-08332-w (PMC11735409; doi:10.1038/s41586-024-08332-w)
Supplement: Supplementary file 2 — Reporting Summary [file 41586_2024_8332_MOESM2_ESM.pdf]

nature portfolio

Corresponding author(s): Dr. James Briscoe

Last updated by author(s): Jun 3, 2024

Reporting Summary

Nature Portfolio wishes to improve the reproducibility of the work that we publish. This form provides structure for consistency and transparency in reporting. For further information on Nature Portfolio policies, see our Editorial Policies and the Editorial Policy Checklist.

Statistics

For all statistical analyses, confirm that the following items are present in the figure legend, table legend, main text, or Methods section.

n/a	Confirmed	
☐	☒	The exact sample size (*n*) for each experimental group/condition, given as a discrete number and unit of measurement
☐	☒	A statement on whether measurements were taken from distinct samples or whether the same sample was measured repeatedly
☐	☒	The statistical test(s) used AND whether they are one- or two-sided<br>*Only common tests should be described solely by name; describe more complex techniques in the Methods section.*
☒	☐	A description of all covariates tested
☒	☐	A description of any assumptions or corrections, such as tests of normality and adjustment for multiple comparisons
☐	☒	A full description of the statistical parameters including central tendency (e.g. means) or other basic estimates (e.g. regression coefficient) AND variation (e.g. standard deviation) or associated estimates of uncertainty (e.g. confidence intervals)
☐	☒	For null hypothesis testing, the test statistic (e.g. *F*, *t*, *r*) with confidence intervals, effect sizes, degrees of freedom and *P* value noted<br>*Give P values as exact values whenever suitable.*
☒	☐	For Bayesian analysis, information on the choice of priors and Markov chain Monte Carlo settings
☒	☐	For hierarchical and complex designs, identification of the appropriate level for tests and full reporting of outcomes
☐	☒	Estimates of effect sizes (e.g. Cohen's *d*, Pearson's *r*), indicating how they were calculated

Our web collection on statistics for biologists contains articles on many of the points above.

Software and code

Policy information about availability of computer code

Data collection	RT-PCR data was acquired using Applied Biosystems QuantStudio software.<br>Immunofluorescence images were acquired using Zeiss Zen (v3.1) or Leica SP8 software.
Data analysis	Fiji v2.14 was used for image analyses, display and formatting. The custom-made pipelines to analyze single-cell data and for image quantifications were deposited on github. The scripts to analyse single-cell transcriptomic data are available https://github.com/tiagu/trunk_dev_scRNAseq. The Nucleus segmentation pipeline is available https://github.com/tiagu/Nucleus and the respective PyTorch models have been deposited in Zenodo (https://zenodo.org/records/11388472). The pipeline to analyse 3D cultures is available https://github.com/tiagu/gastrunet (DOI 10.5281/zenodo.12684779).<br>Imaris v9.5.1 was used for volumetric measurements and lightsheet movie creation.<br>The following packages were used for analyses: CellRanger (v4.0.0), Scanpy (v1.7.0), Scrublet (v0.2.2), harmonypy (v0.0.10), SAMtools (v1.9) dropEst (v0.8.6).

For manuscripts utilizing custom algorithms or software that are central to the research but not yet described in published literature, software must be made available to editors and reviewers. We strongly encourage code deposition in a community repository (e.g. GitHub). See the Nature Portfolio guidelines for submitting code & software for further information.

Data

Policy information about [availability of data]

All manuscripts must include a [data availability statement]. This statement should provide the following information, where applicable:

- Accession codes, unique identifiers, or web links for publicly available datasets
- A description of any restrictions on data availability
- For clinical datasets or third party data, please ensure that the statement adheres to our [policy]

Single-cell 3' mRNA sequencing data (10X Genomics) have been deposited under accession numbers GSE223189, GSE224404 and GSE255338 for chick trunk, human micropatterns and 3D notoroids respectively. Human data were mapped to the GRCh38-3.0.0 reference genome.
The following data series were re-analysed GSE122187 (mouse transcriptomics; mm10) and GSE193007 (cynomolgus monkey).
EM data is deposited in the Electron Microscopy Public Image Archive with the EMPIAR IDs: 12161,12162, 12163 and 12164.

Human research participants

Policy information about [studies involving human research participants and Sex and Gender in Research.]

Reporting on sex and gender	NA
Population characteristics	NA
Recruitment	NA
Ethics oversight	NA

Note that full information on the approval of the study protocol must also be provided in the manuscript.

Field-specific reporting

Please select the one below that is the best fit for your research. If you are not sure, read the appropriate sections before making your selection.

☒ Life sciences ☐ Behavioural & social sciences ☐ Ecological, evolutionary & environmental sciences

For a reference copy of the document with all sections, see nature.com/documents/nr-reporting-summary-flat.pdf

Life sciences study design

All studies must disclose on these points even when the disclosure is negative.

Sample size	Sample size and depth of single-cell experiments were based on previous experience gained during the studies of Delile et al. 2019 and other similar studies. For qRT-PCR assays all samples collected over the course of exploratory and validatory stages of the study were aggregated for statistical analysis. For the remaining experiments no statistical test was performed to predetermine sample size.
Data exclusions	Melt-curve analysis and extreme cT values were used as a basis for exclusion outliers of qPCR technical replicates prior to statistical analysis of data. In micropatterned culture, pattens that were merged or not well isolated from their surroundings neighbors were excluded.
Replication	Reported results were repeated and confirmed in at least three independent experiments, except for single-cell RNA sequencing of embryos which was performed at closely-timed stages of development. For single-cell experiments of micropatterns and trunk-organoids, each experiment was reproduced and performed with multiple biological and/or technical replicates as stated in the Methods section.
Randomization	No particular randomization method was used in this work. For trunk organoid experiments, either the entire plate per experiment per condition was assayed or a given proportion of the plate was arbitrarily picked to be assayed (minimum n=8).
Blinding	Blinding was not performed in this study as the experimental setup and observation involves direct handling of the samples, e.g. application of treatments or cell counting.

Reporting for specific materials, systems and methods

We require information from authors about some types of materials, experimental systems and methods used in many studies. Here, indicate whether each material, system or method listed is relevant to your study. If you are not sure if a list item applies to your research, read the appropriate section before selecting a response.

Materials & experimental systems

n/a	Involved in the study
☐	☒ Antibodies
☐	☒ Eukaryotic cell lines
☒	☐ Palaeontology and archaeology
☐	☒ Animals and other organisms
☒	☐ Clinical data
☐	☒ Dual use research of concern

Methods

n/a	Involved in the study
☒	☐ ChIP-seq
☒	☐ Flow cytometry
☒	☐ MRI-based neuroimaging

Antibodies

Antibodies used

The primary antibodies used in this study are detailed below:
mouse SOX2-AF488 BDBiosciences #561593 1:100
goat SOX2 R&D AF2018 1:500
rabbit TBXT D2Z3J #81694 1:500
goat TBXT R&D AF2085 1:1000
goat TBX6 R&D AF4744 1:500
TJP1 (ZO-1) Thermofisher #61-7300 1:200
goat SOX1 R&D AF3369 1:1000
rabbit FOXA2 Seven Hills WRAB-1200 1:2000
rabbit Phospho-p44/42 MAPK (Erk1/2) Cell Signalling 9101S 1:200
mouse total YAP Santa Cruz 63.7 (sc-101199) 1:100
rabbit active YAP Abcam EPR19812 (ab205270) 1:100
goat SNAIL R&D AF3639 1:500
rabbit LEF1 Cell Signalling #2230 1:200
goat SOX17 R&D AF1924 10ug/ml
goat HAND1 R&D AF3168-SP 1:200
mouse SHH DSHB 5E1 1:500
goat OLIG2 R&D AF2418 1:1000
sheep FOXC2 R&D AF5044 1:500
mouse NKX6.1 DSHB F55A10 1:100

Secondary antibodies (1:1000):
AF488 Donkey anti-mouse Invitrogen Cat#A21202
AF488 Donkey anti-goat Invitrogen Cat#A11055
AF488 Donkey anti-rabbit Invitrogen Cat#A21206
NL557 Donkey anti-Goat RnDsystems Cat#NL001
NL557 Donkey anti-Rabbit RnDsystems Cat#NL004
NL557 Donkey anti-Mouse RnDsystems Cat#NL007
AF594 Donkey anti-rabbit Invitrogen Cat#A32754
AF647 Donkey anti-mouse Invitrogen Cat#A31571
AF647 Donkey anti-goat Invitrogen Cat#A21447
AF647 Donkey anti-rabbit Invitrogen Cat#A31573
AF647 Donkey anti-sheep Invitrogen Cat#A21448
WGA iFluor™ 488 STEMCELL Technologies Cat#100-0816
DAPI Cell Signaling Technology Cat#4083S

Validation

All antibodies are commercial and have been validated by the companies from which they were purchased from in addition to being highly referenced in the field across several laboratories. Please see a list below of the main antibodies employed in this study. Furthermore, antibodies were tested for possible unspecific signals at unrelated embryonic stages or in vitro control conditions. Only antibodies with a reproducible signal were used.

goat SOX2 R&D AF2018; IF validation in ADLF1 and FAB2 Stem Cell Lines; 352 references.
rabbit TBXT D2Z3J #81694; IF validation in MUG-CHOR-1 and MCF 10A cell lines; 32 references.
goat TBXT R&D AF2085; IF validation in BG01V and differentiated human stem cells as well as mouse notochord; 240 references.
goat TBX6 R&D AF4744; IF validation in Mouse Mesoderm and JOY6 iPS cells ; 19 references.
goat SOX1 R&D AF3369; IF validation in Human Developing Brain, differentiated BG01V and NTera-2 cell lines; 127 references.
rabbit Phospho-p44/42 MAPK (Erk1/2) Cell Signalling 9101S; IF validation in ; 32 references.
rabbit active YAP Abcam EPR19812 (ab205270); IF validation in HUVEC and 293A ; 85 references.
goat OLIG2 R&D AF2418; IF validation in human brain and oligodendrocytes in Rat Cortical Stem Cells ; 191 references.

Eukaryotic cell lines

Policy information about cell lines and Sex and Gender in Research

Cell line source(s)	Human ESCs from the H9 line were obtained directly from WiCell and MasterShef4 from UKSCB. HEK293T cell line was obtained and authenticated by HPA cultures.
Authentication	WiCell database. Karyotyping was performed to verify genome integrity.
Mycoplasma contamination	All cell lines used in this study were routinely (every 3months) screened for Mycoplasma spp. and tested negative.
Commonly misidentified lines (See ICLAC register)	No lines from this register were used in the study.

Animals and other research organisms

Policy information about studies involving animals; ARRIVE guidelines recommended for reporting animal research, and Sex and Gender in Research

Laboratory animals	Fertilized hens' eggs were obtained from Henry Stewart & Co. Ltd.
Wild animals	NA
Reporting on sex	Sex information in the transcriptomic data of chicken embryos was addressed by excluding genes in the W chromosome.
Field-collected samples	NA
Ethics oversight	Chicken embryos were incubated to less than two thirds of their gestation therefore do not fall under the UK's Animals (Scientific Procedures) Act 1986.

Note that full information on the approval of the study protocol must also be provided in the manuscript.

Dual use research of concern

Policy information about dual use research of concern

Hazards

Could the accidental, deliberate or reckless misuse of agents or technologies generated in the work, or the application of information presented in the manuscript, pose a threat to:

No	Yes	
☒	☐	Public health
☒	☐	National security
☒	☐	Crops and/or livestock
☒	☐	Ecosystems
☒	☐	Any other significant area

Experiments of concern

Does the work involve any of these experiments of concern:

No	Yes	
☒	☐	Demonstrate how to render a vaccine ineffective
☒	☐	Confer resistance to therapeutically useful antibiotics or antiviral agents
☒	☐	Enhance the virulence of a pathogen or render a nonpathogen virulent
☒	☐	Increase transmissibility of a pathogen
☒	☐	Alter the host range of a pathogen
☒	☐	Enable evasion of diagnostic/detection modalities
☒	☐	Enable the weaponization of a biological agent or toxin
☒	☐	Any other potentially harmful combination of experiments and agents
