# Supplementary material for: Timely TGFβ signalling inhibition induces notochord
Source: Nature. 2024 Dec 18;637(8046):673–82. doi: 10.1038/s41586-024-08332-w (PMC11735409; doi:10.1038/s41586-024-08332-w)

nature portfolio

https://doi.org/10.1038/s41586-024-08332-w

Supplementary information

Timely TGFβ signalling inhibition induces notochord

In the format provided by the authors and unedited

Supplementary Fig. 1 - Single-cell RNA-seq of chick trunk development: combined embedding of all stages.
a, Integrated UMAP embedding of chick trunk (4-13 Somites) coloured by Leiden clustering (resolution parameter=3.5) used to form the final clustering of trunk populations in Fig.1b. b, proportions of cell types captured at stages of 4S-13S of chicken development. c, contribution of the individual stages to the main UMAP of main Fig.1b and Fig.S1a. d, Force-directed graph embedding showing the presence of the main trunk populations at each individual stage. The plots highlight that in all stages the same populations can be found as expected for caudal elongation stages. e-g, UMAP showing the expression of key genes characterizing the PXM and PS; LPM and intermediate mesoderm; and notochord and endoderm populations found in the chick trunk (4S-13S).

Supplementary Fig. 2 - Single-cell RNA-seq of chick trunk development: additional cell type markers.
a-b, Integrated UMAP embedding of chick trunk (4-13S) showing the expression of several genes marking the surface ectoderm; the preneural, neural and neural crest populations present during chick trunk formation. c, Gene expression of anterior preneural genes identified by Trevers et al. (2021). Scale with maximum counts is indicated by the squares.

Supplementary Fig. 3 – Single-cell RNA-seq data of mouse and macaque trunk formation.
a, Integrated UMAP embedding of mouse trunk formation (E8.0-E8.5) from Chan et al. (2019). Gene expression of main markers for each trunk population. b, UMAP showing the expression of key genes characterizing the Primitive streak; Lateral Plate Mesoderm (LPM), notochord, NMPs, endoderm and intermediate mesoderm populations found in the mouse trunk. Unbiased Leiden clusters used to define main trunk populations. c, Equivalent embedding of macaque trunk formation (CS9-CS11) from Zhai et al. (2022) coloured by the main trunk populations. Gene expression of main markers for each trunk population and the Leiden clusters used to define them.

Supplementary Fig. 4 – Cell population markers during chick trunk formation.
a, Optical section of a 7 somite chicken embryo HCR stained for NEFM shows localization in the caudal portion of somites and in the mature neural plate border. b, Immunostaining of SOX1 highlighting the first SOX1+ cells in rostral part of the sinus rhomboidalis, immediately rostral and on the side of the median pit. Arrows highlight the caudal beginning of the expression domain. c, HCR of a 7S chicken embryo stained for ADAMTS18, CYP26A1, TBX6 and CNTN2. Blue arrows represent the ventral axial border of TBX6 expression.

Supplementary Fig. 5 – Mouse neural-mesodermal fate decision and TGFb signaling inhibitors in chick trunk populations.
a, Transcriptional similarity ordering (pseudotime analysis) of mouse single cell data showing gene expression of several lineage and signalling genes. Cells corresponding to PXM, NMPs and Neural tissue were taken from clusters 9,8,2,11 (Fig.S3b Leiden clustering). b, Pie charts with percentage of cells (red) in each cell population expressing at least one BMP or NODAL inhibitor.

Supplementary Fig. 6 - *Nucleus* cell segmentation pipeline.
a, Overview of the *Nucleus* pipeline: cascade R-CNN is used for object detection in each z-plane, 2D nuclei are aggregated in 3D nuclei models which are further refined by a correction step. b, (left table) Training datasets used to train and validate the neural network. (right) Histogram with the maximum nuclear lengths in each dataset. These can be used to guide image acquisition as to whether the network will perform as expected. (bottom) Performance table example showing how different architectures predict the validation set by different average precision metrics. c, Examples of 2D segmentations performed by this tool.

Supplementary Fig. 7 – Cell populations around the posterior end node.
a, HCR of a 7S chicken embryo stained for CHRD, SPRY2, FOXA2 and LEFTY1. b, Top view (maximum intensity projection) around the node. (*right*) Dorso-ventral optical sections and quantification of HCR signal along the midline. The plot shows a spline fitted to the relative gray values for the different channels in the rostrocaudal orientation (x-axis, left to right)   c, Immunostaining showing TBXT and FOXA2 co-localization at the median pit. R and C stand for rostral and caudal direction in the embryo.

Supplementary Fig. 8 – Spatial mapping to chicken embryo: from HCR levels to position and from position to HCR data.
a, Scatter plot of HCR signals per single cell for NOTO, CNTN2 and NEFM in 7S chicken embryos; colouring reflects agglomerative hierarchical clustering (Ward's Linkage) with Euclidean distance where the linkage distance threshold was chosen to yield 5 clusters. Clusters were spatially mapped back to the embryo: cells with high NOTO and high CNTN2 HCR signals map to the medial pit of the 7S embryo (red cells in right sections). b, 3D centroids given by the Nucleus pipeline were selected by choosing a rectangle (x-, y-position) at a given dorso-ventral location (z-position). Dorsal and ventral positions around the median pit where picked and overlayed on a DAPI image. HCR cell levels of NOTO and CNTN2 are plotted in red for the cell cohorts of the selected positions. c, Solovieva et al. (2022; Fig.3) analysed long-term resident cells of the posterior node (HH3+/4 to HH8 end-point). A, M, P stands for the anterior, middle and posterior HH8 node subregions. Re-analysis of gene expression of CHRD, CNTN2, LEFTY2 and SPRY2 showed the expected high CHRD levels for the cells in all positions and just the anterior node has high LEFTY2. These data also indicate the posterior node cells at HH8 have higher CNTN2 and SPRY2 levels.

Supplementary Fig. 9 – Posterior neuruloid.
a, Monolayer differentiation of NMPs as in Figure 3a stained for SOX2 and TBXT. Nuclear levels of both markers showing a broad range of levels. b, Maximum intensity projection showing size-dependant organization of TBXT+ tissue (red) for colony diameters between 200-500µm. Red line shows the optical section used in main figure 3a. c, Posterior neuruloid micropattern colonies of different size with the MasterShef4 cell line.

Supplementary Fig. 10 – Posterior neuruloid at 12h post-induction.
a, Immunostaining overview of a micropatterned coverslip stained for pERK1/2 of posterior neuruloids at 12 hours post-induction. b, Staining of pERK1/2 and TBXT induction with different FGF concentrations, MEK inhibition and with no WNT agonist (CHIR; just N2B27+FGF).

Supplementary Fig. 11 – TRULI-treated posterior neuruloid.
a, Characterization of the TRULI-treated neuruloid showing no TBX6 or FOXA2 cells at day3. b, Morphology of TRULI-treated neuruloid at day3 highlighting lower TBXT induction at the top and centre of the colonies with only a few edge cells staining positive.

Supplementary Fig. 12 – YAP signaling and TBXT induction in neuruloids and chicken embryos.
a, YAP stains at 12 and 48h post-induction. Colonies at different stages (12, 24 and 48h) were 3D segmented and the number of cells with different YAP and TBXT levels quantified. TBXT high cells were tested for presence of YAP and significance was assessed by a Chi-square test of independence separately for each time-point. Conversely, we also analysed YAP high cells for the presence of high TBXT levels showing YAP high cells disproportionally have low TBXT levels at all time-points. b, Immunostaining of a chicken embryo at head process stage (HH5) for active YAP and TBXT. *(right)* DAPI and TBXT zoomed out image. (*left*) Panels show several z-planes from ventral to dorsal embryo

zoomed around the node (yellow box on right panel). TBXT and active YAP together and active YAP alone are displayed for each z-plane. High-TBXT cells are accompanied by a down-regulation of active YAP. This is further showed by the cross-section (note high active YAP cells at the neural tube edges and endoderm). 3D segmentation of the embryo also shows TBXT+ segmented cells lie at the low end of the active YAP levels distribution for all cells in the embryo.

Supplementary Fig. 13 – Characterization of delayed inhibition conditions.
a, Immunostaining of SOX17 and FOXA2 in a 48h delayed inhibition neuruloid showing their co-expression in the same cell population. b, Direct-force graph embedding layout with the integrated transcriptomic data of posterior neuruloid together with the 24h and 48h delay in TGFβ inhibitors protocol variations. Gene expression of additional lateral plate mesoderm marker genes.

Supplementary Fig. 14 – Cell type classifier across species.
a, Confusion matrix for the random forest classifier trained on chick and mouse embryo cell clusters identified during trunk formation. A high recovery is observed for each cell population and the overall accuracy is 77% (with 25% data held for testing). 166 genes representing the intersection of highly variable genes across species were used to train the classifier with 5000 trees. b, Random Forest classifier predictions with macaque and human cell queries. Macaque and human embryo *in vivo* cell populations, where we have higher assignment confidence, were used to independently validate the classifier before comparing with human cells from posterior neuruloids and notoroids. The different clusters were obtained from the combined cells across all BMP and NODAL inhibition delay treatments, e.g. the endoderm cell cluster is only present in the 48h treatment whilst notochord cells are mostly present in the 24h delay treatment.

Supplementary Fig. 15 – Single-cell RNA-seq markers of trunk-organoids.
a, 2D embeddings of notoroid single-cell data showing genes expressed in the notochord population, including negative/ low (at day 3) GSC expression characteristic of the early node when it acts as organizer. b, Embeddings showing binary information of single cells co-expressing (or not) combinations of different genes.

Supplementary Fig. 16 – Characterization of different inhibition delays using imaging data.
a, Experimental design of notoroids with different inhibition delays of main Figure 6f with delays every 6h from 12-36h post-induction. b, GastrUnet was trained with FOXC2 and TBXT immunofluorescence images to estimate the 3D proportion of somitic mesoderm and notochord from 14 z-planes per notoroid. c, Examples of elongation in the 18h and 36h delay inhibition conditions. (*right*) single z-plane of a 36h delay notoroid showing a large FOXC2+ population and also many TBXT+ cells. d, Estimated fraction of FOXC2 and TBXT per notoroid by delay in inhibition. Although both increase with delay, the FOXC2 proportion does so faster than TBXT. e, Solidity of brightfield images showing the lowest values for the 18h delay condition, these are associated with higher trunk-organoid areas (*right*).

Supplementary Fig. 17 – Dorso-ventral neural cell types in notoroid using scRNA-seq.
a, 2D embeddings of notoroid single-cell data showing binary information of single cells co-expressing combinations of different genes characteristic of neural progenitor domains in the dorsal (PAX6+PAX3+, only at day5) and ventral spinal cord (p2-3, pMN and FP). PAX7+ cells are seen in the Floor plate cells, a transcriptome signature previously observed in human but not in mouse (Rayon et al. 2021).

Supplementary Fig. 18 – Analyses of notoroids with TNE-ZsGreen H9 cell line.
a, ZsGreen channel signal of notoroids with the TBXT Notochord-Enhancer (TNE)-ZsGreen H9 reporter cell line versus a no enhancer (no CRE) control (n=48). b, Unprocessed 96-well plates images of notoroids with the TNE-ZsGreen reporter line with and without vismodegib SHH antagonist. c, Output of the GastrUnet pipeline to analyse brightfield and ZsGreen images. The magenta outline represents the main trunk-organoid mask selected from the image. The main filament path used to calculate maximum length is displayed in red. A conservative estimation of just the bright ZsGreen signals is shown in dark green outlines – see histogram of these ZsGreen areas in main Figure 5k. The magenta dots represent estimated filament end-points which were extended to the nearest trunk-organoid edge to give a more accurate measure of its maximum length. d, To measure the maximum ZsGreen intensity at the midline along the notoroid length, successive areas were taken along the notoroid main filament (red line in panel c). e, Pearson correlation of several morphometrics with the percentage of pixels ZsGreen+ of the trunk-organoid (px_ON_perc_gfp_organoid). Interestingly, higher ZsGreen area correlates with lower trunk-organoid areas, higher solidity and lower max lengths. This suggests trunk-organoids with lower ZsGreen were more similar to the 18h delay condition. Note these notoroids all have 24h delay inhibition and all these values are estimations of the 3D structure from 2D brightfield images.

Supplementary Fig. 19 – Characterization of notochord in notoroids by immunostaining.
a, Stain with phalloidin and wheat germ agglutinin (WGA), a known conventional marker for the Golgi apparatus and vesicles/ early endosomes, indicates TBXT+ cells have abundant Golgi vesicles and a relatively large cytoplasm. b, TBXT+SOX9+ co-expression. Note SOX9 is also expressed at low levels in neural and somitic mesoderm cells.

Supplementary Fig. 20 – Main ultrastructural features found in the notochord of notoroid.
Desmosomes (purple arrows), cilia and microvilli (pink arrows), dilated ER (green arrows), ECM fibers and vacuoles (cyan arrows).

Supplementary Fig. 21 – Distinct ultrastructural features of neural and mesenchymal cells.
Low resolution detail of neural and mesenchymal cells in the notoroids highlighting their distinct morphology from notochordal cells.

Supplementary Fig. 22 – Scanning Electron Microscopy of freeze-fractured notoroids.
A, Low magnification SEM image of a freeze-fractured trunk-organoid. B-C) Higher magnification SEM images of ECM deposition. D-F) Higher magnification SEM images of a putative notochord. Lipid droplets are visible (yellow stars). Neuroepithelium cells (n) are indicated. Both neural and notochordal regions are covered by a basal lamina and surrounded by a sheath of fibres (yellow arrows). Regions of interest shown in higher magnification images are denoted with colour boxes. G-H, High magnification SEM images of the surface of the notoroid. Cilia and microvilli are visible (magenta arrows). Scale bars: 50 µm (A), 20 µm (B, D), 10 µm (C, E), 3 µm (F,G,H).

Supplementary Fig. 1

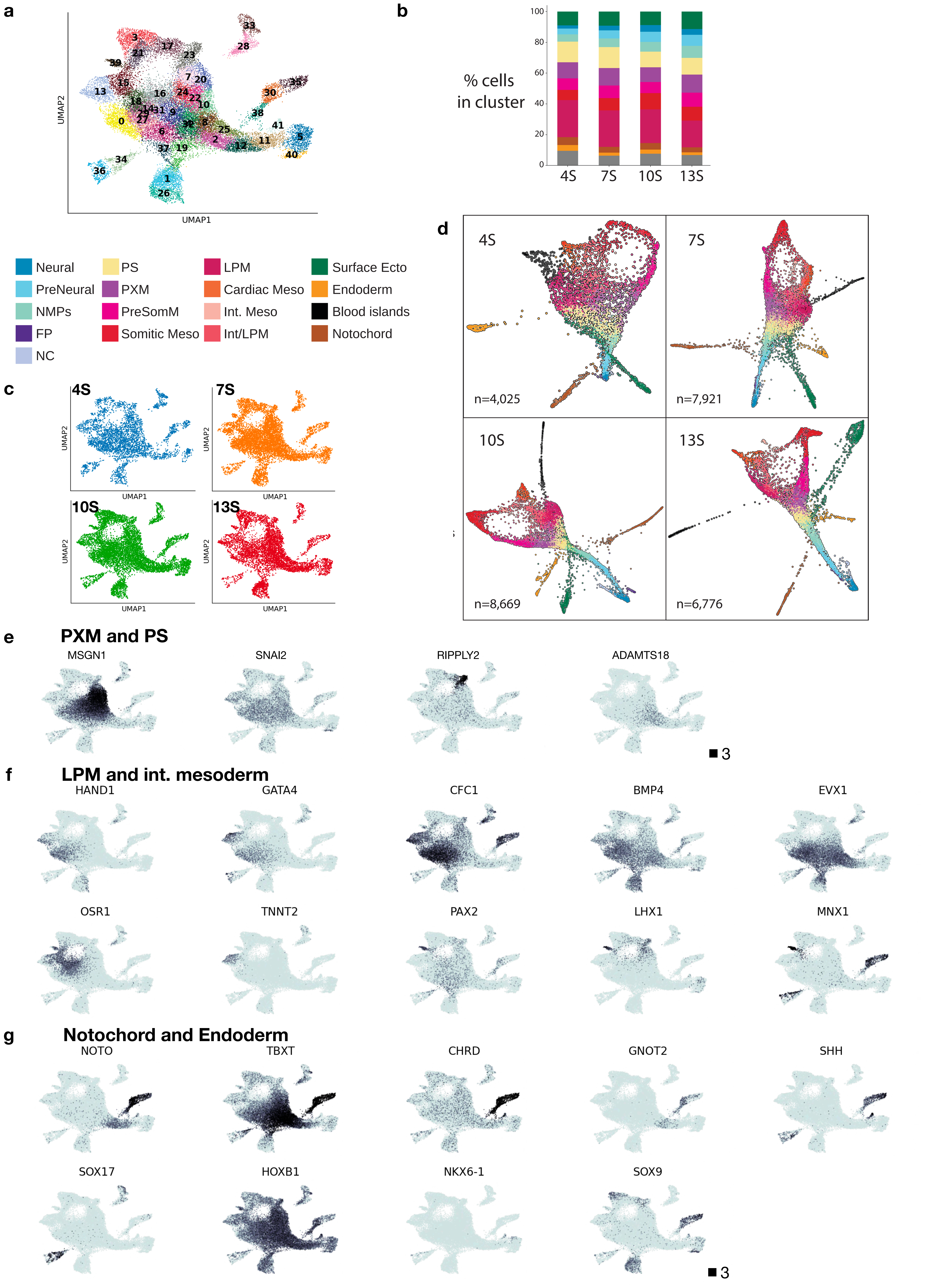

Supplementary Fig. 2

a Surface Ectoderm

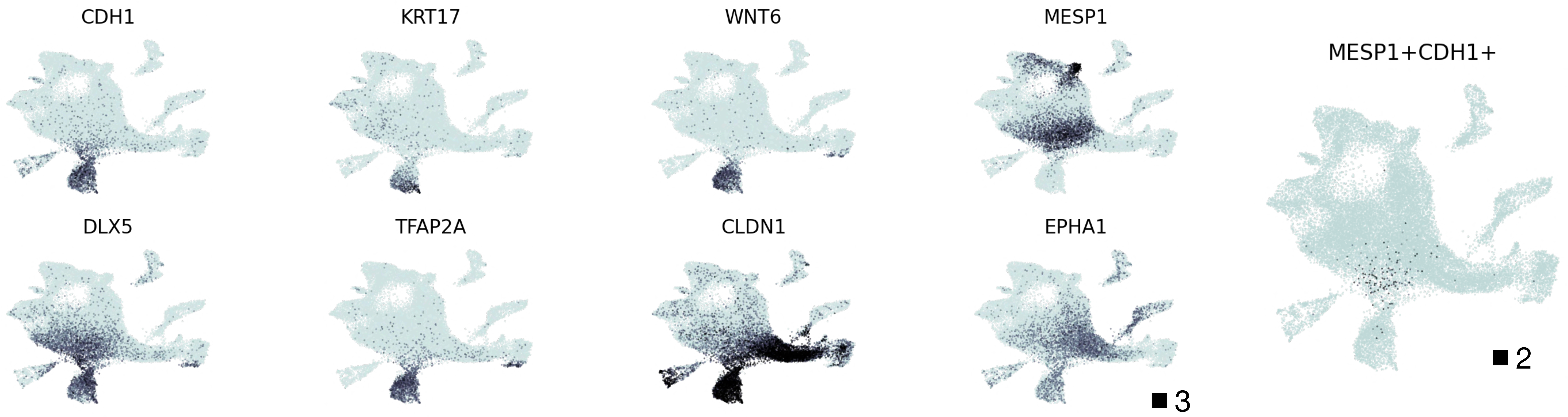

b PreNeural, Neural and Neural Crest

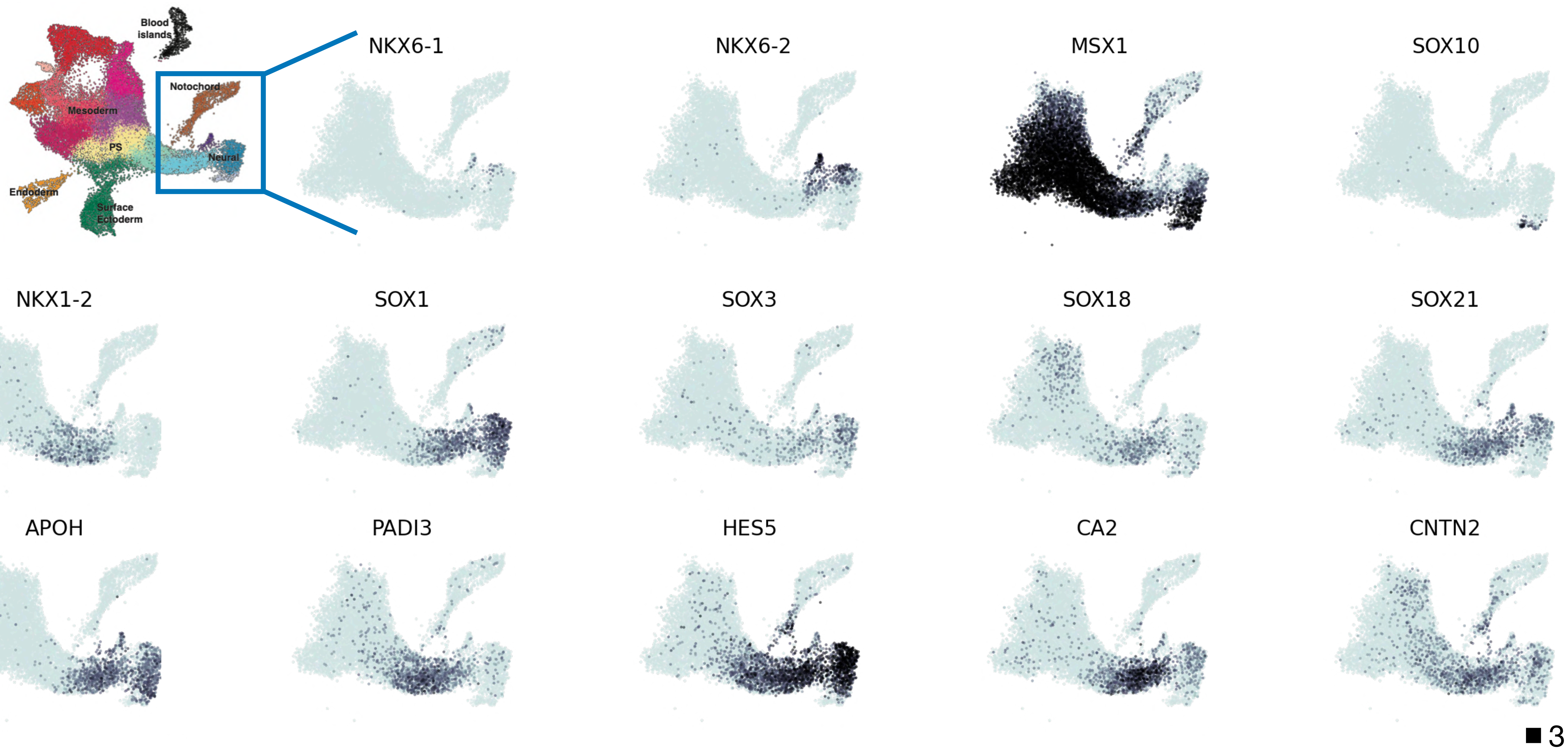

c

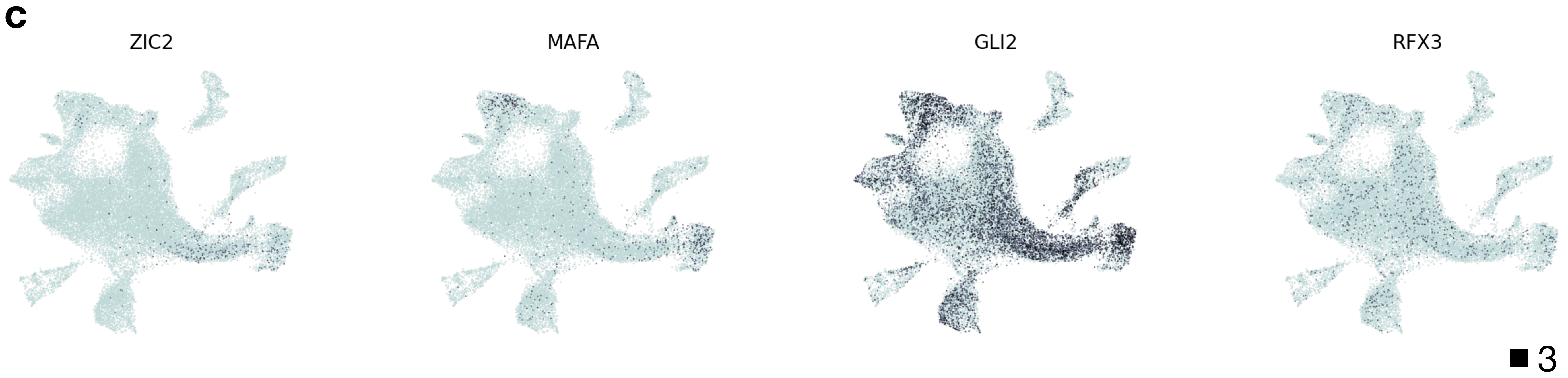

Supplementary Fig. 3

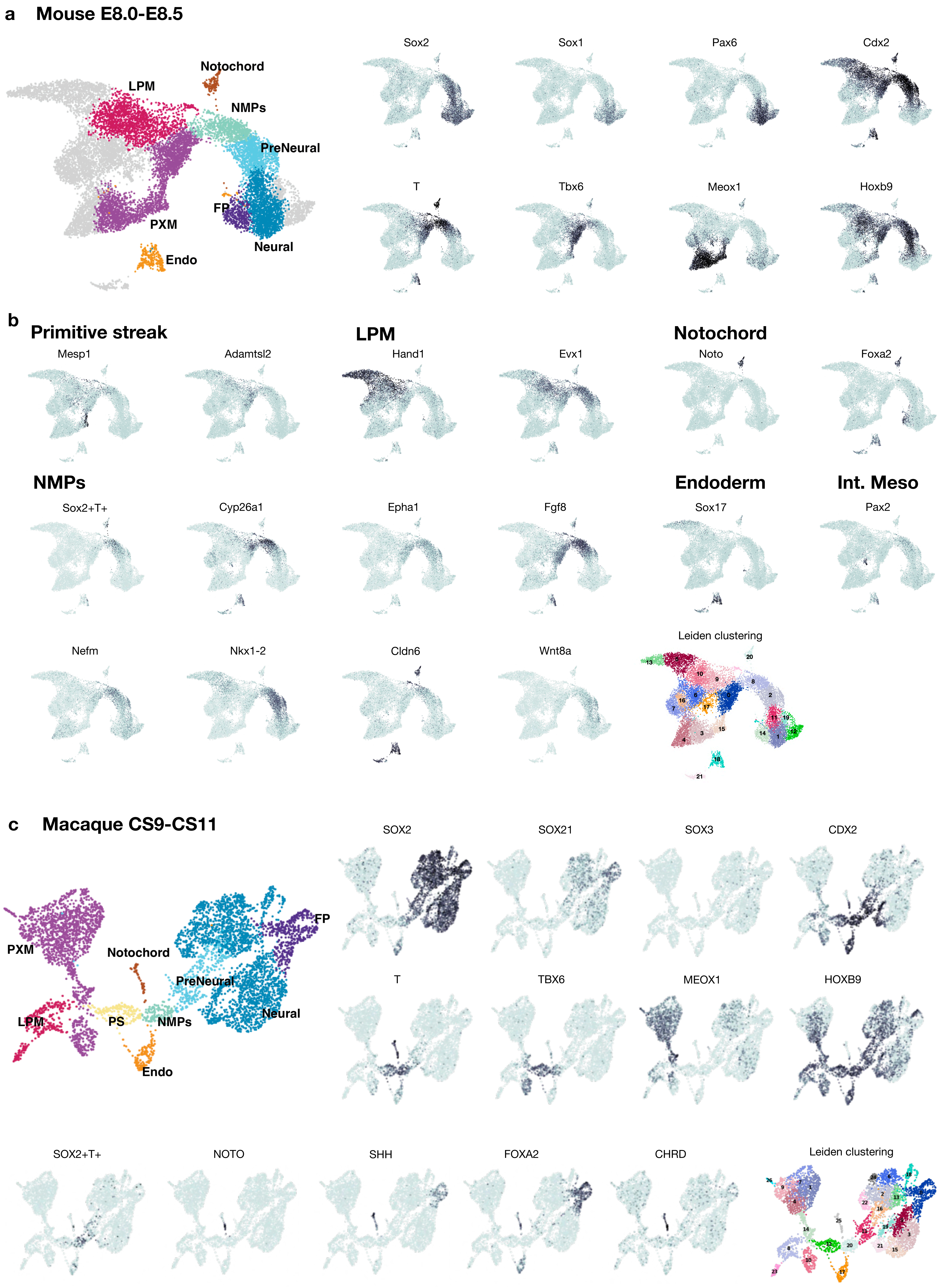

Supplementary Fig. 4

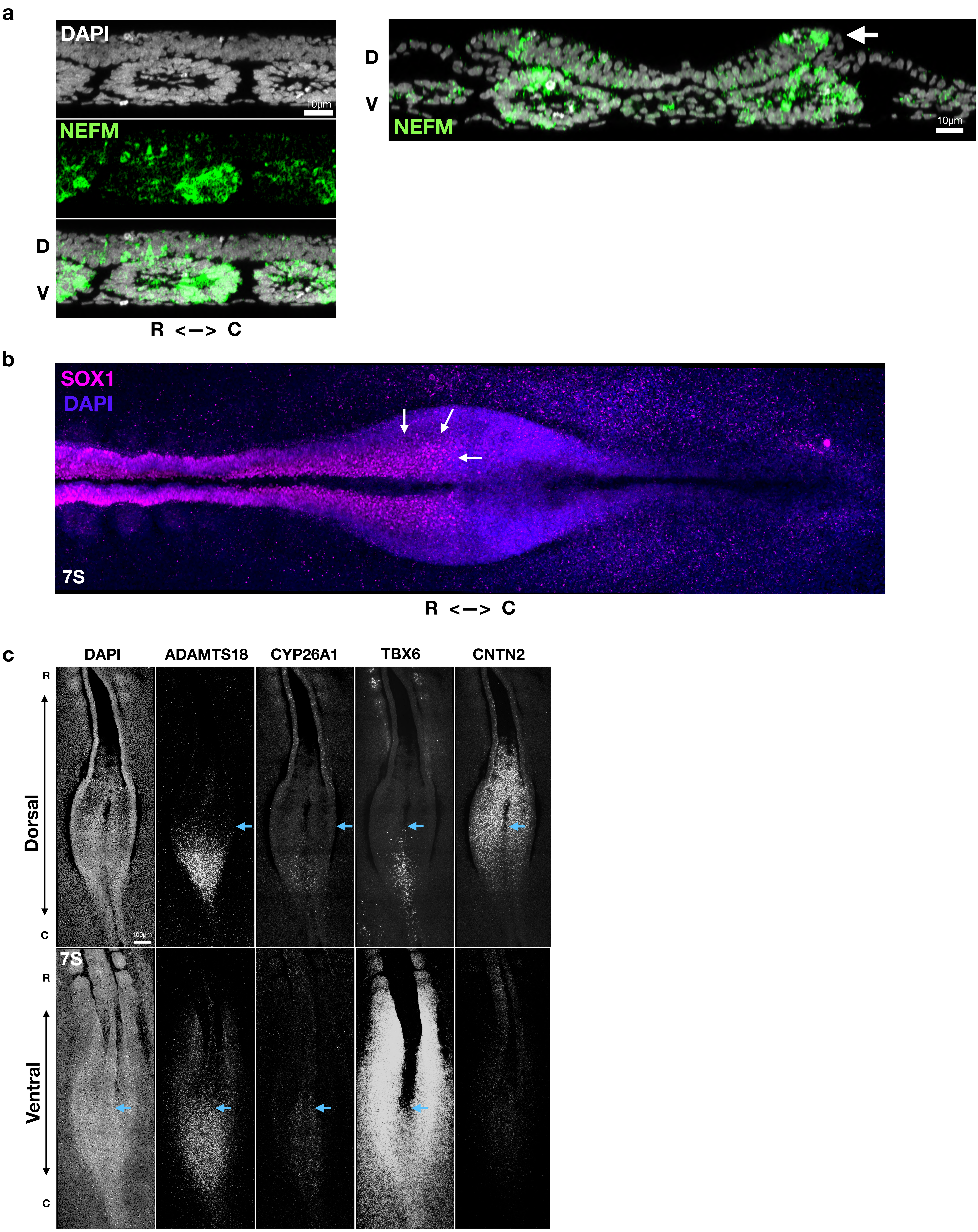

Supplementary Fig. 5

a

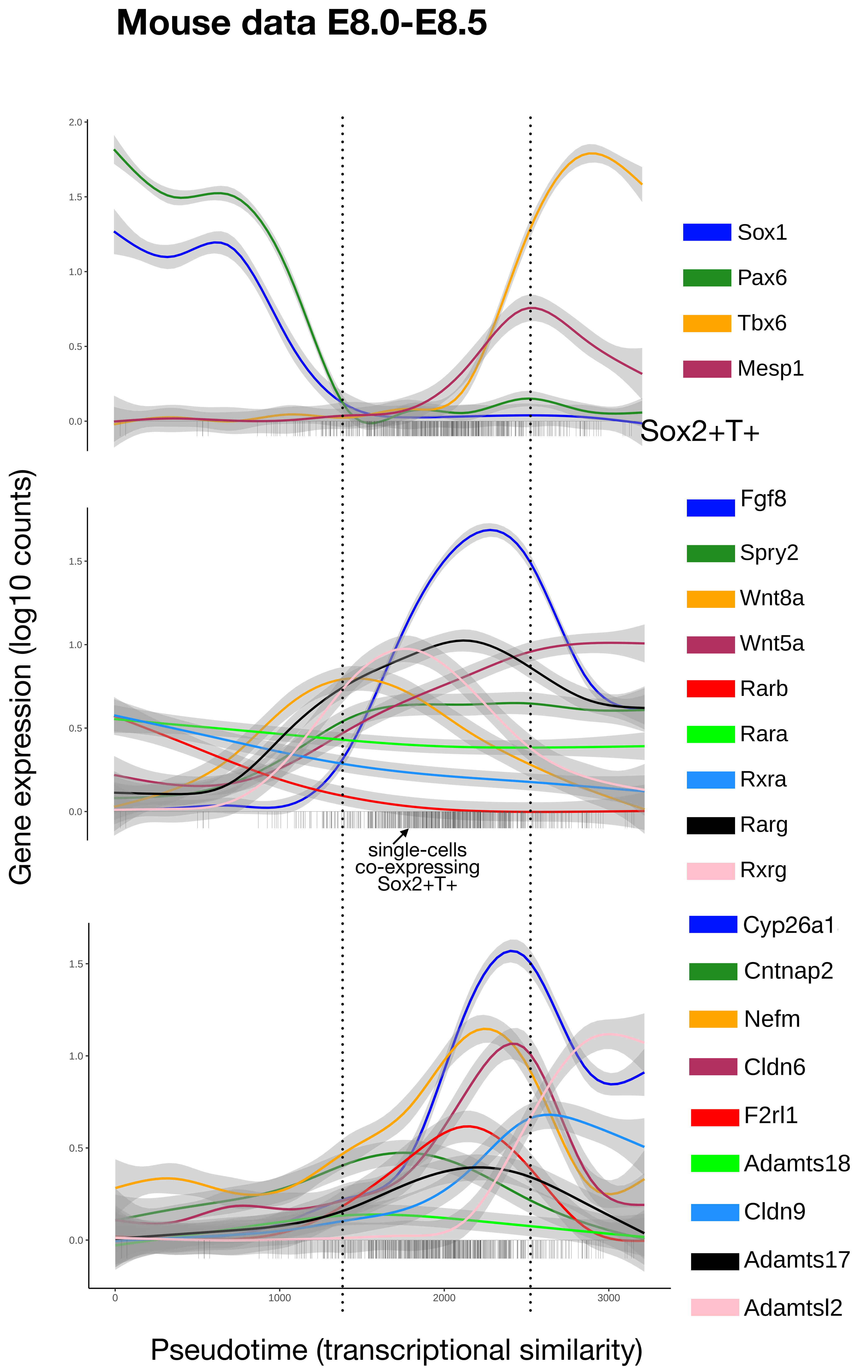

b

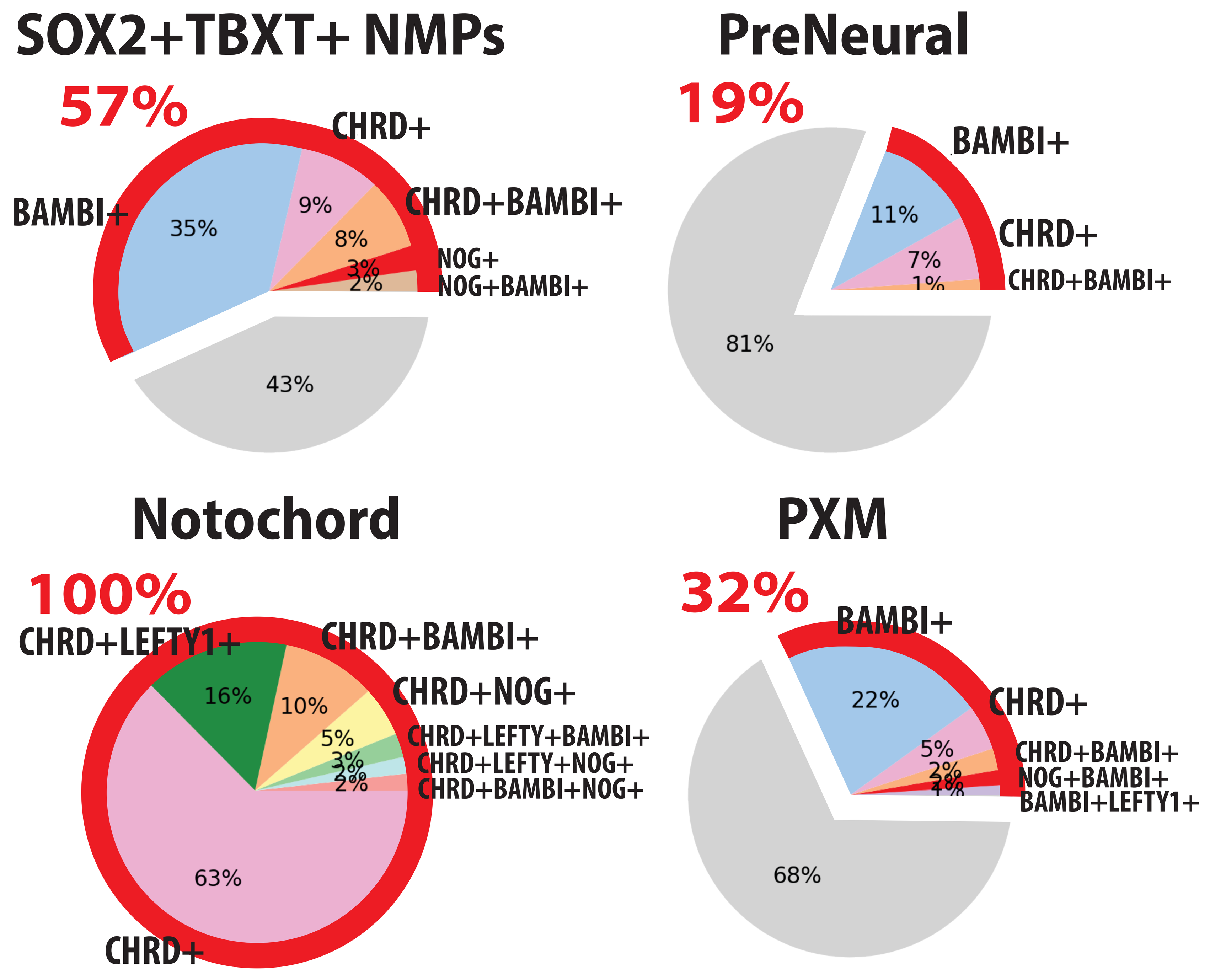

Supplementary Fig. 6

a *Nucleus* pipeline for single-nuclei detection in dense colonies/ embryos

1. High-confidence detection of 2D nuclei in single-z plane using CNN

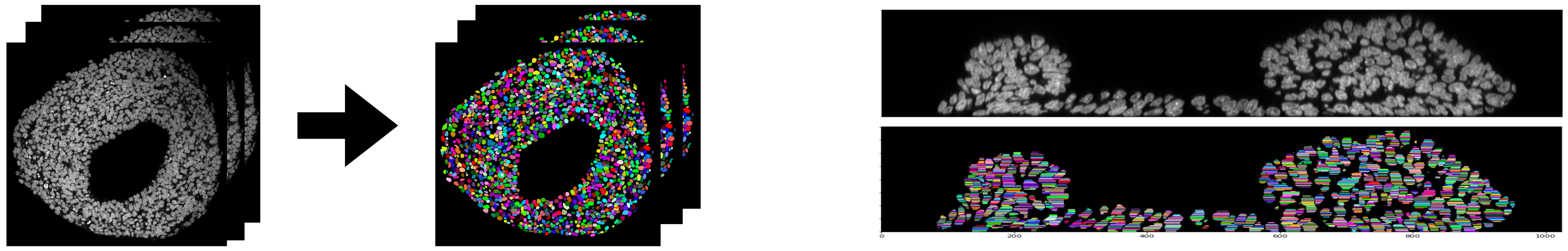

AP50 (avg precision at IoU.50) = 82-86%

2. 3D consolidation and correction

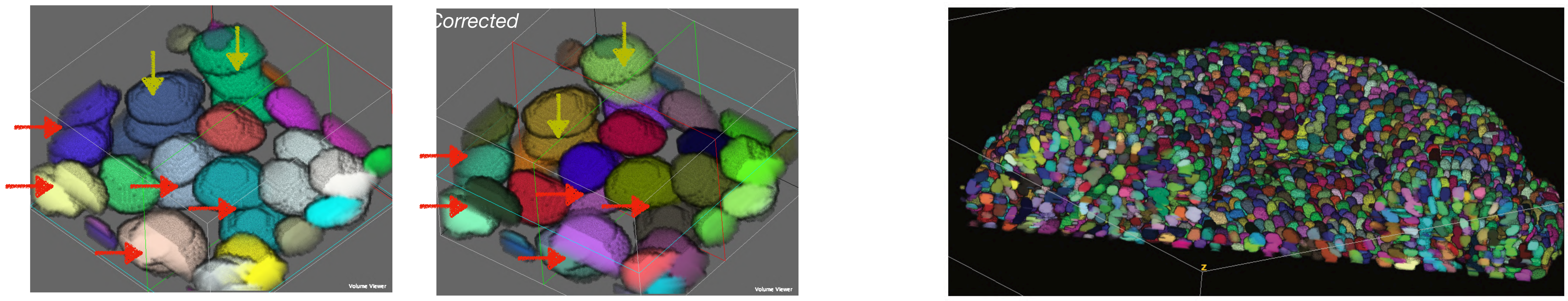

b Training set

	#images	#instances	size	comments
nucleus_train	6	221	256*256	in vitro hESC assay *
nucleus_val (validation)	4	141	256*256	in vitro hESC assay * *
kromp_ 2019	52	1,704	640*512	curated from Kromp et al. (2019)
segm_512	3	566	512*512	in vitro hESC assay *
SC_human	4	X	256*256	Spinal Cord sections *
SC_mouse	6	X	256*256	Spinal Cord sections *
SC_sections (validation)	5	X	256*256	Spinal Cord sections * *

* validation datasets
* new datasets manually segmented

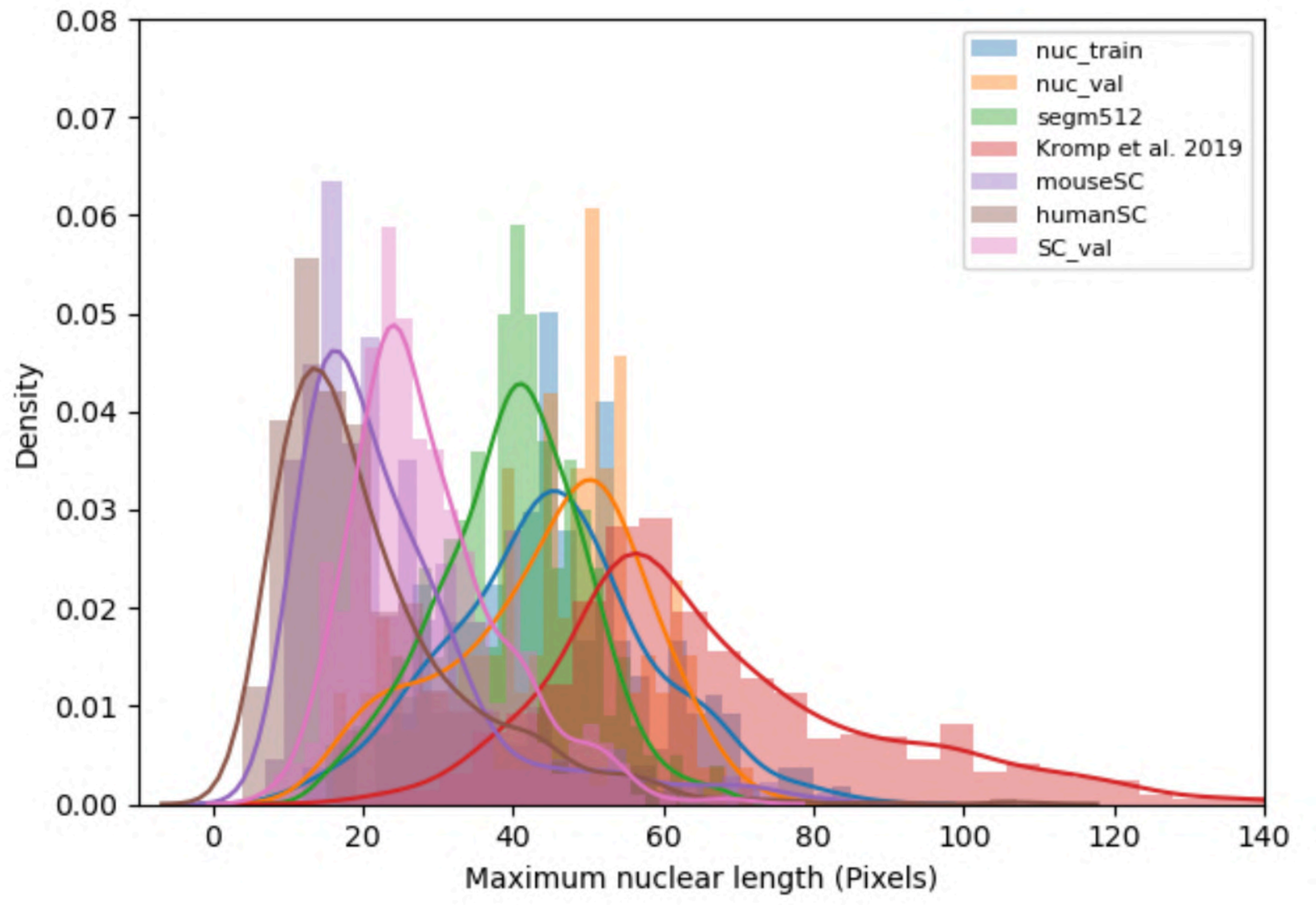

			AP	AP50	AP75	APs	APm	AR
cascade_mask_rcnn_X_152	SC_val	BBOX	44.29	68.21	50.96	45.55	14.12	50.30
		SEGM	42.59	67.31	47.68	42.72	40.00	48.60
	nucleus_val	BBOX	55.19	86.34	63.89	45.52	62.53	59.20
		SEGM	53.11	86.55	61.16	43.87	60.02	57.40
mask_rcnn_X_101	SC_val	BBOX	53.04	81.56	59.58	53.64	90.00	59.00
		SEGM	53.89	82.52	61.08	53.83	90.00	59.10
	nucleus_val	BBOX	50.63	82.83	57.15	42.54	57.21	56.70
		SEGM	49.54	86.29	54.32	42.65	54.92	55.00

c 2D segmentation examples

mouse hindbrain section

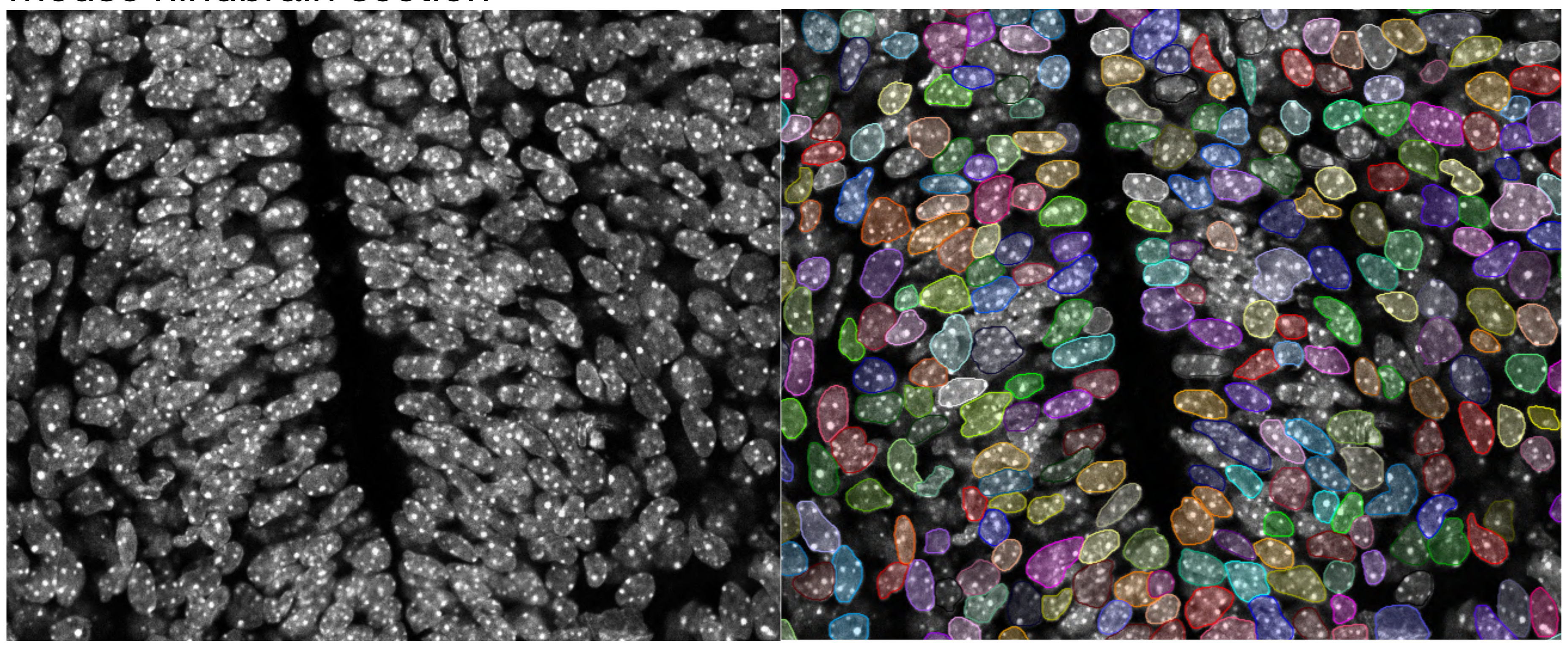

human spinal cord section

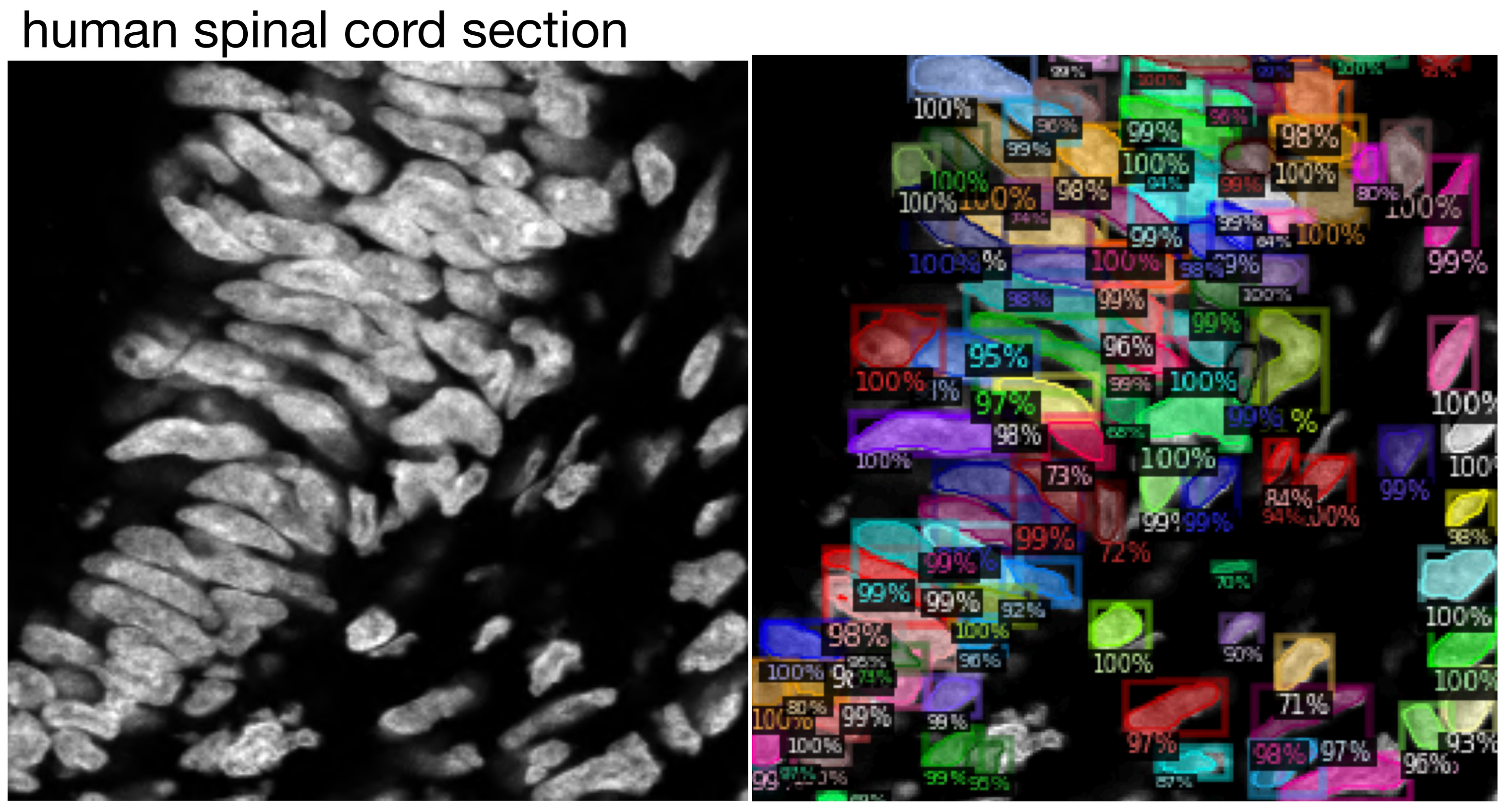

human in vitro organoid

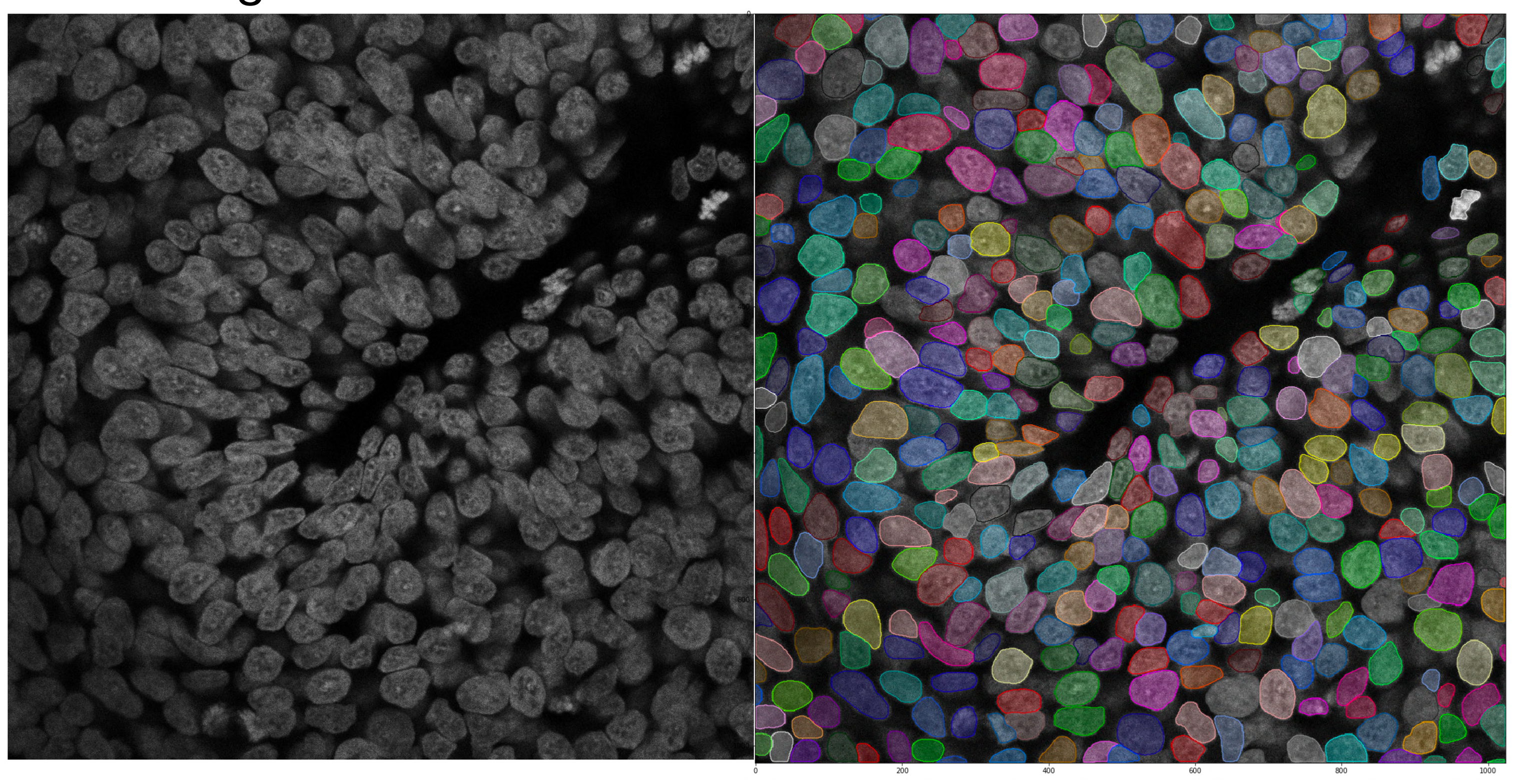

chicken embryo confocal optical section

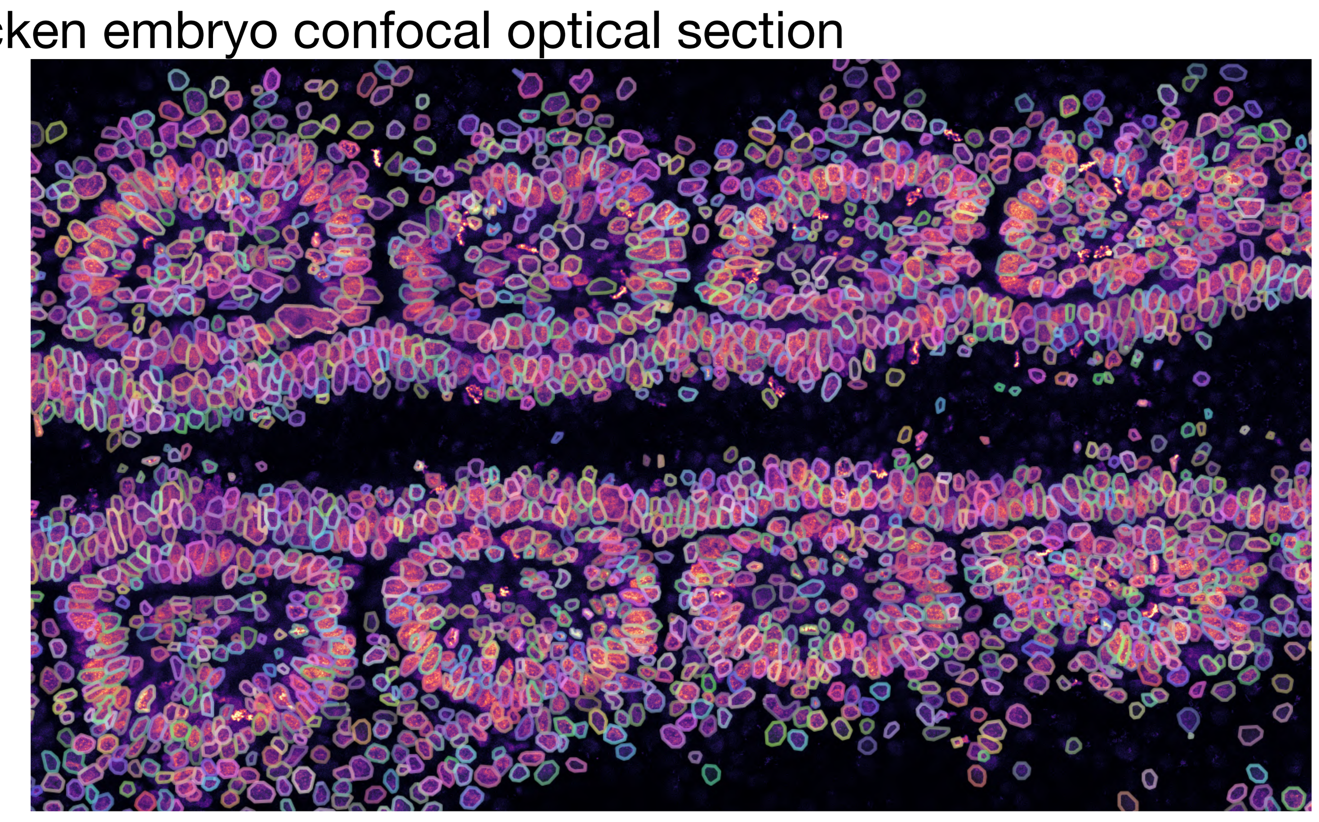

Supplementary Fig. 7

a

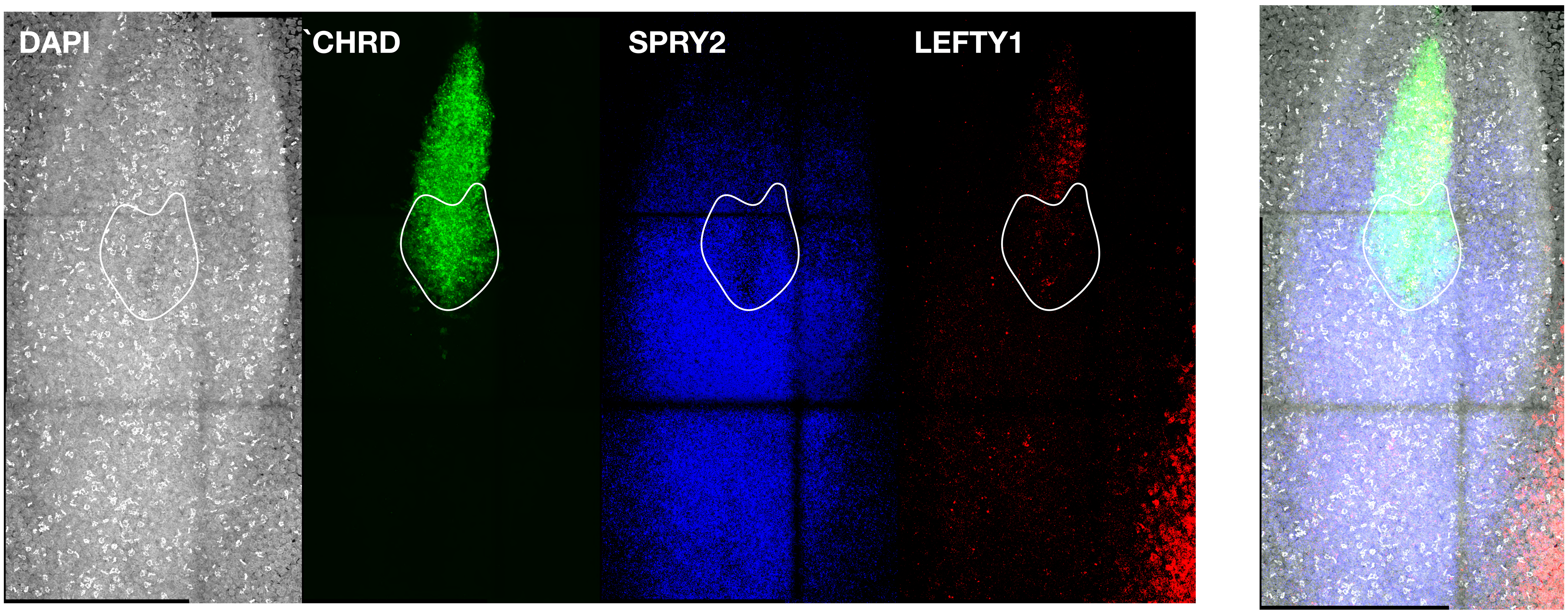

b

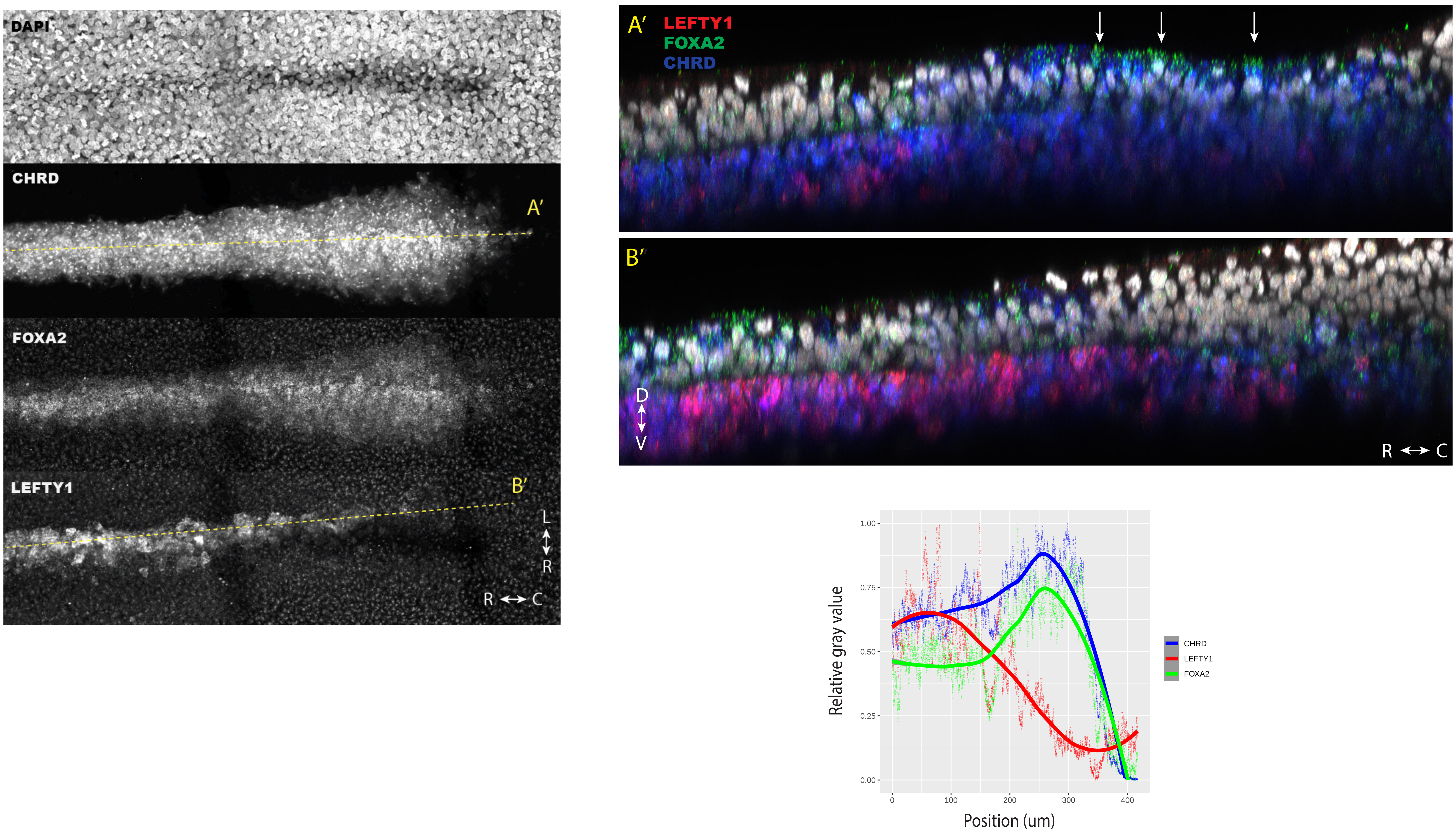

c

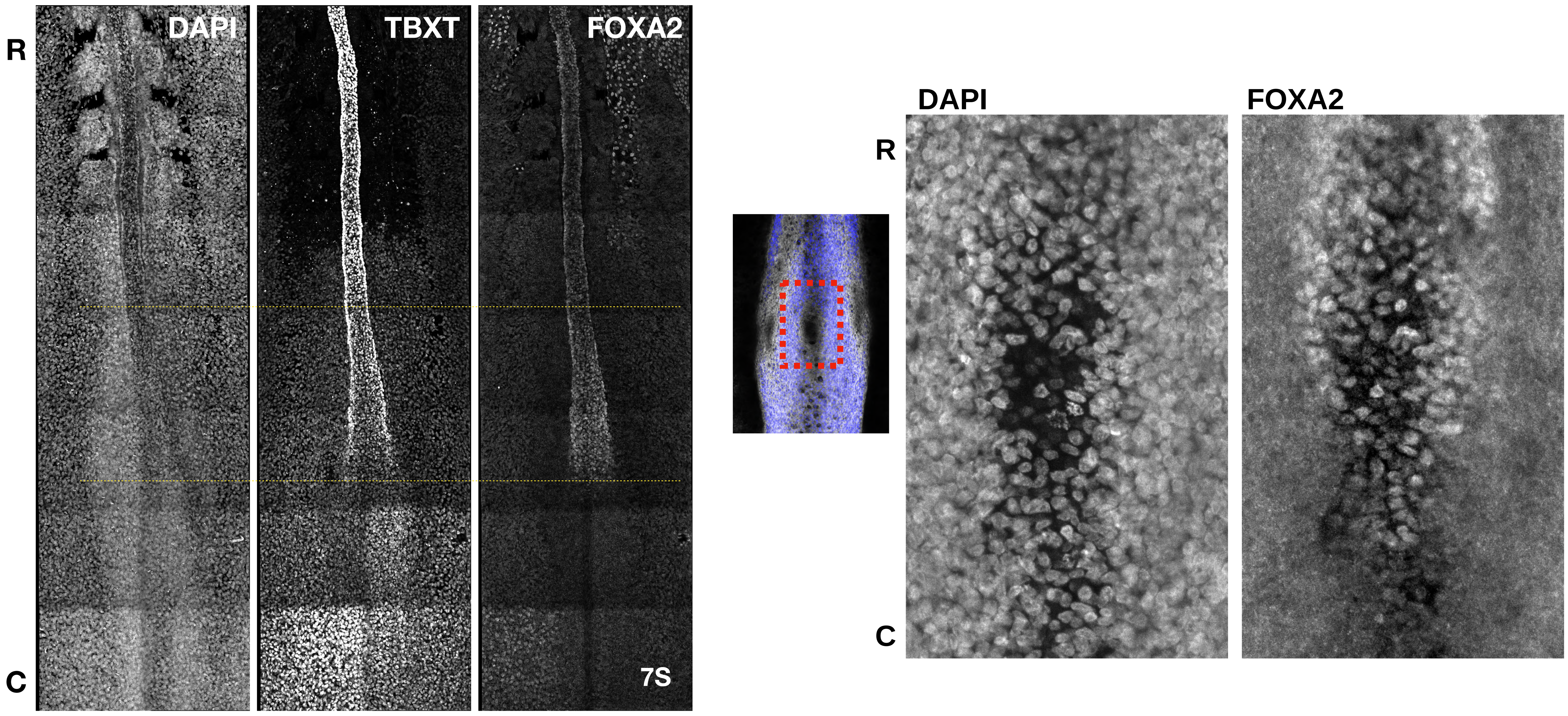

Supplementary Fig. 8

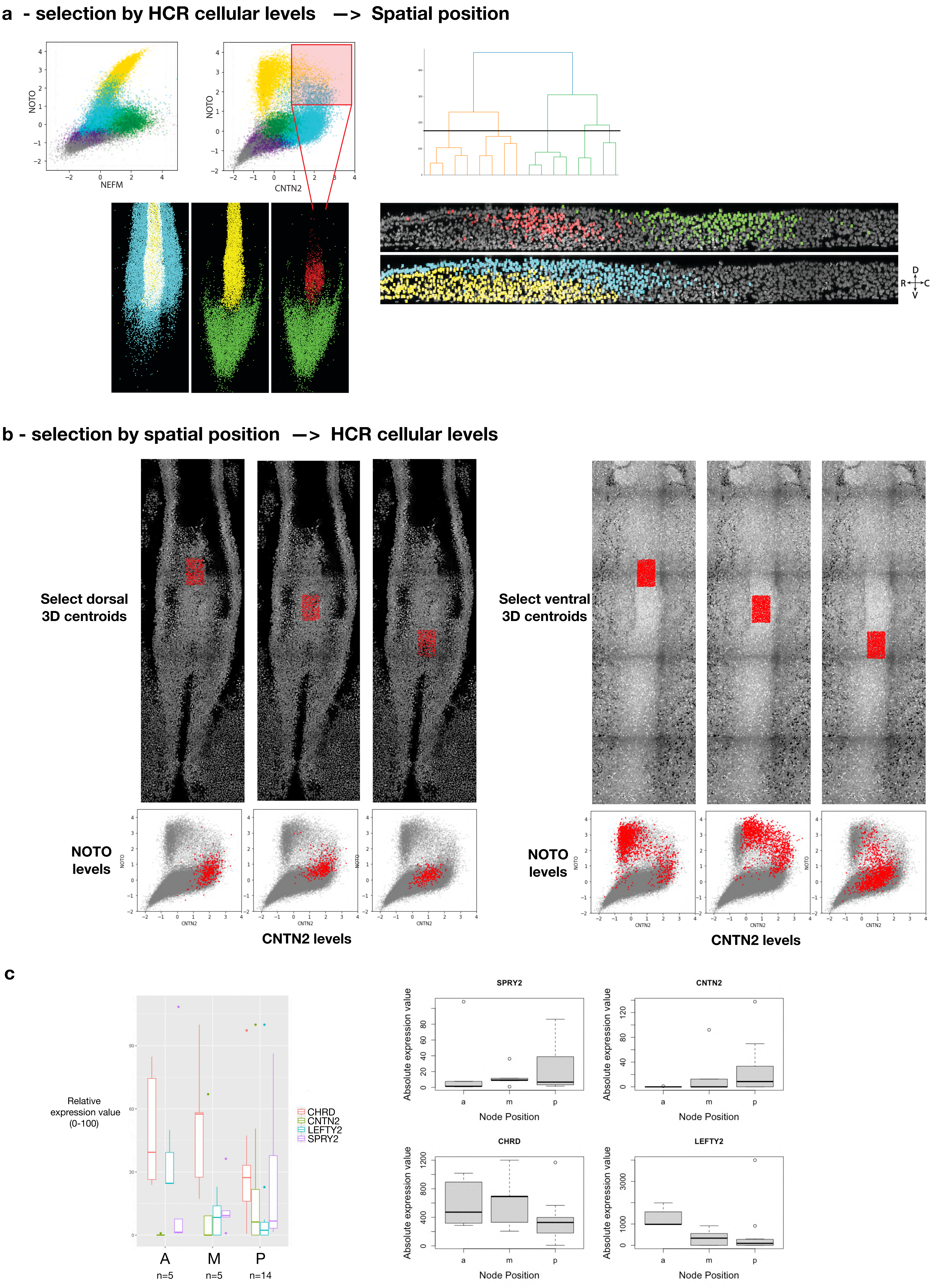

Supplementary Fig. 9

a

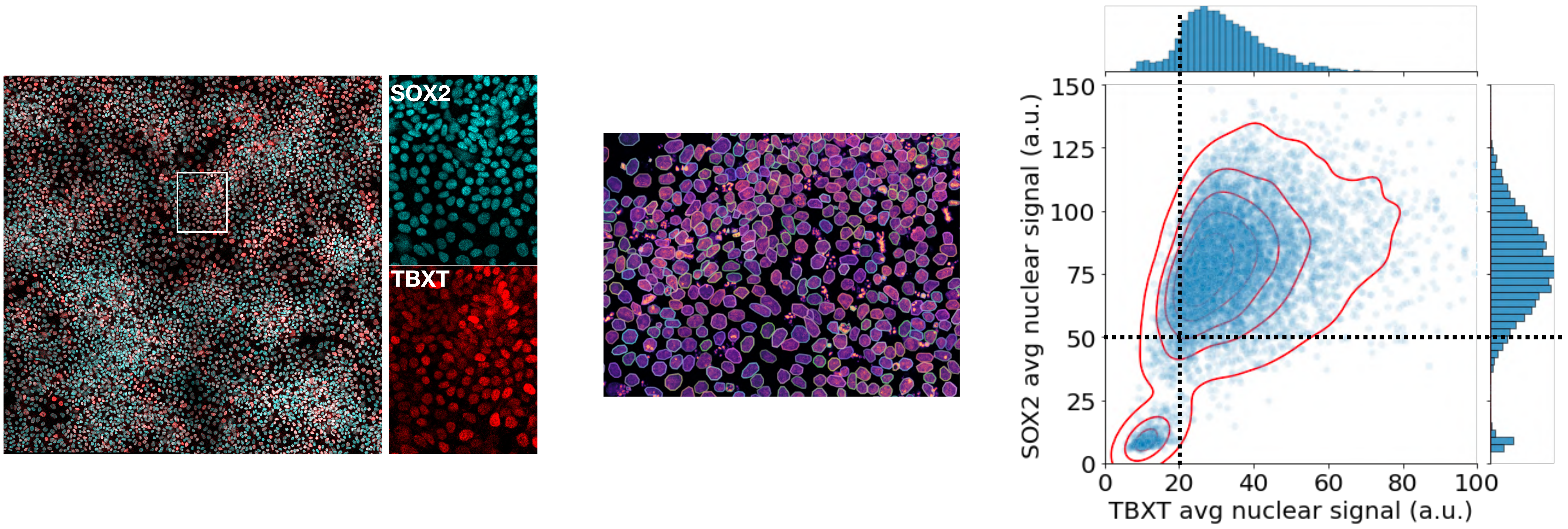

b

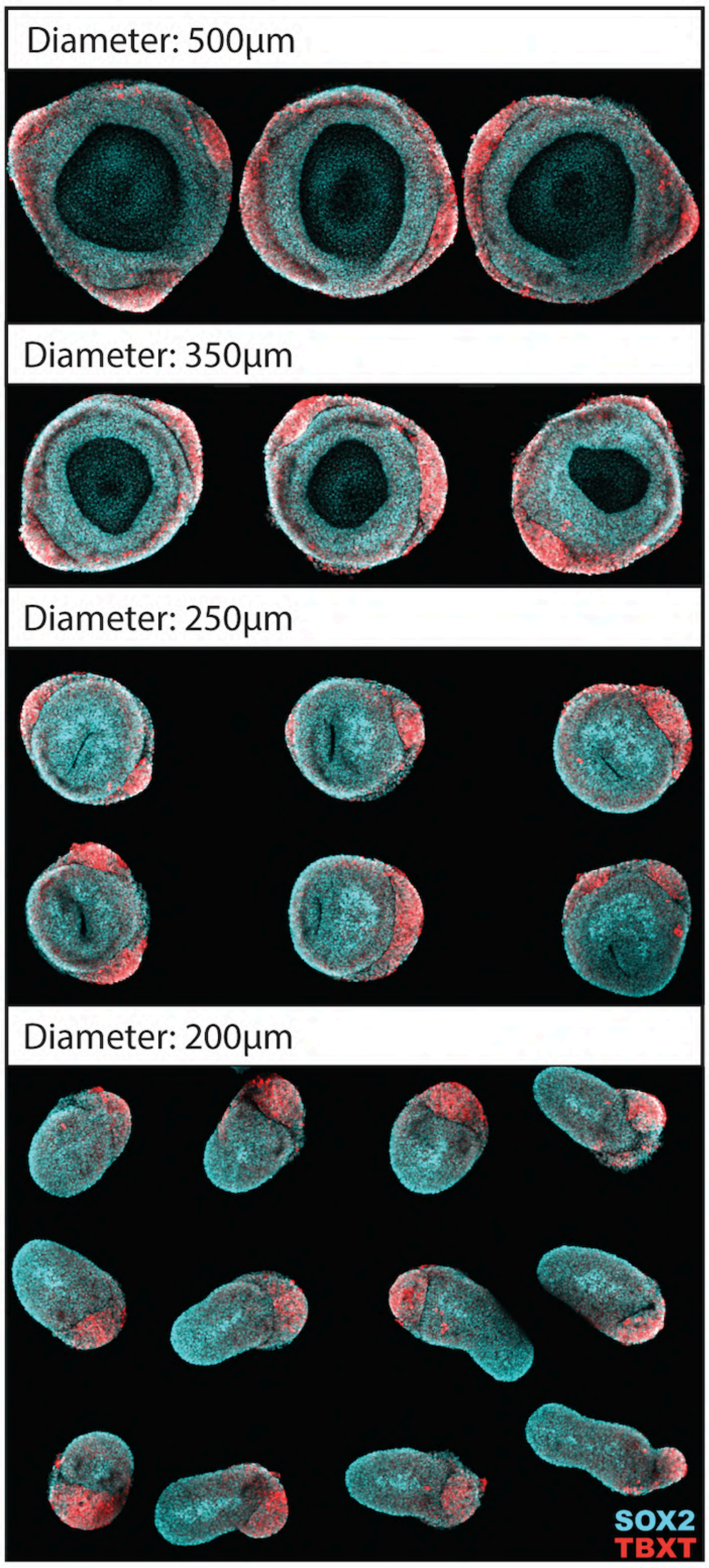

c

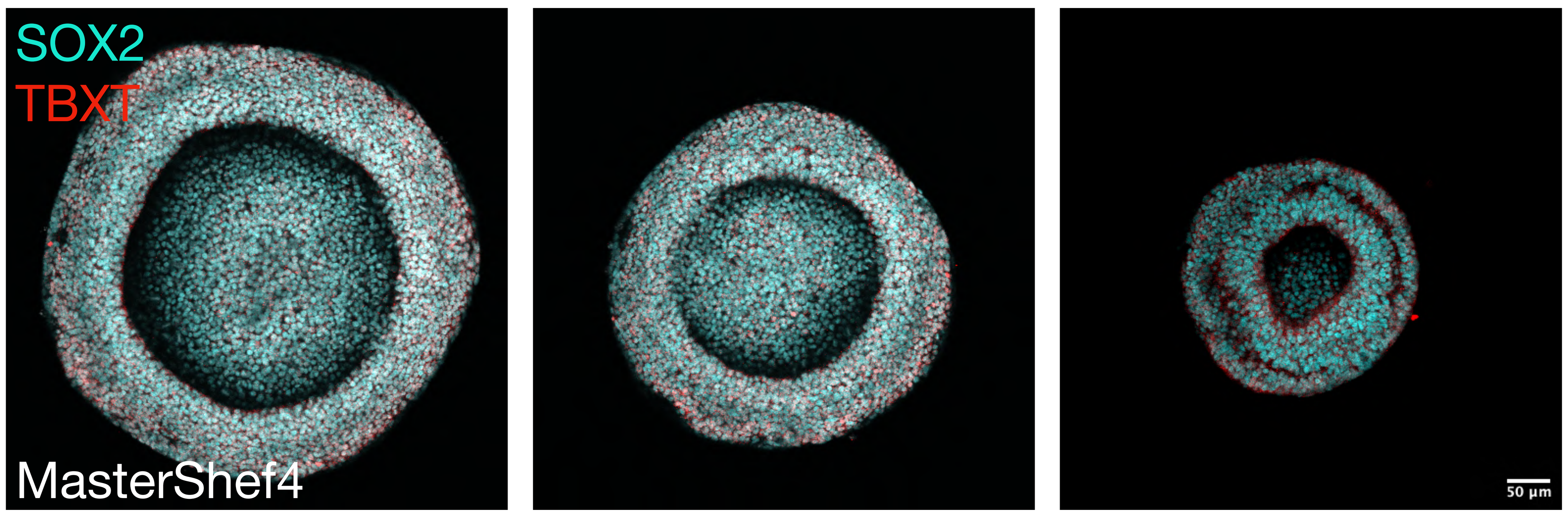

Supplementary Fig. 10

a

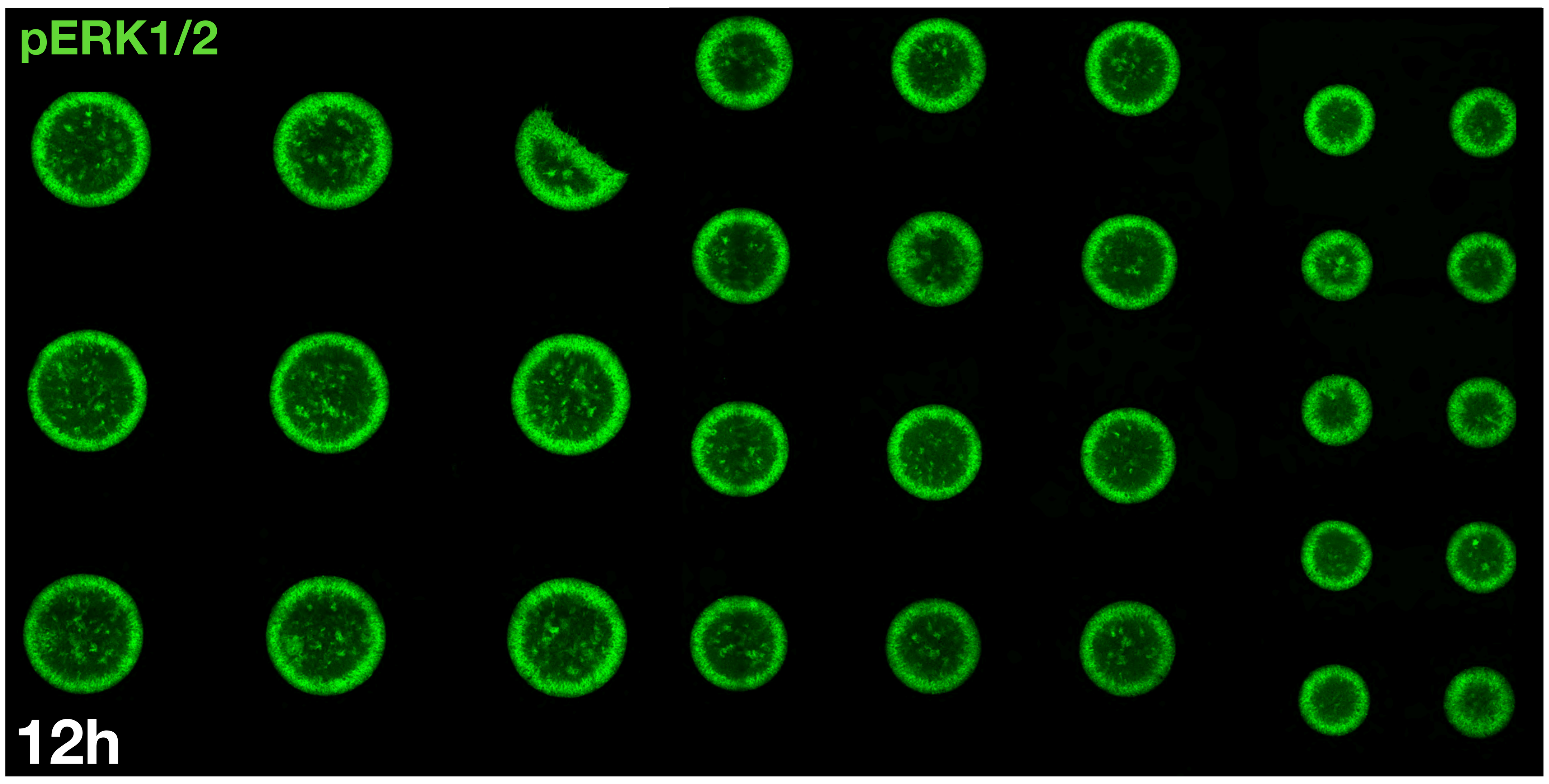

b

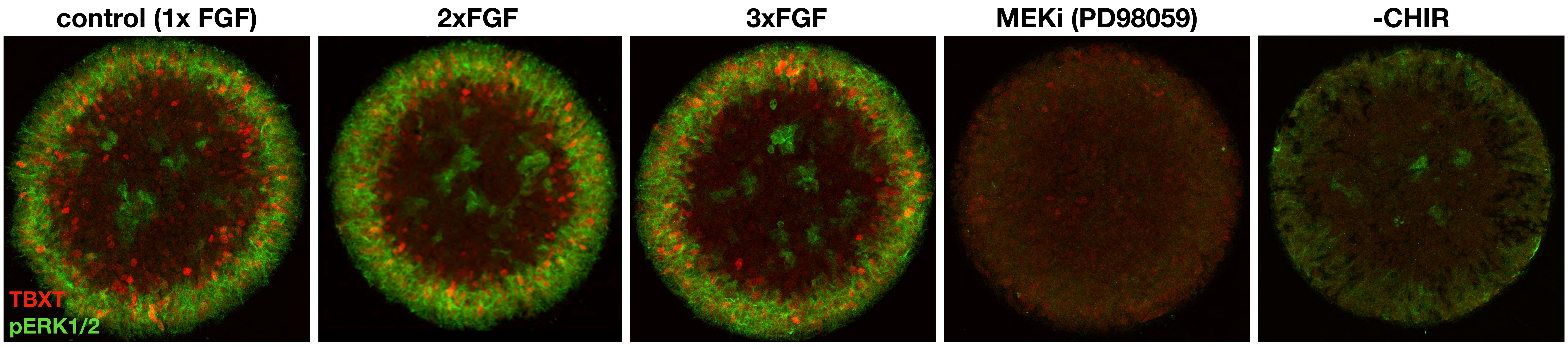

Supplementary Fig. 11

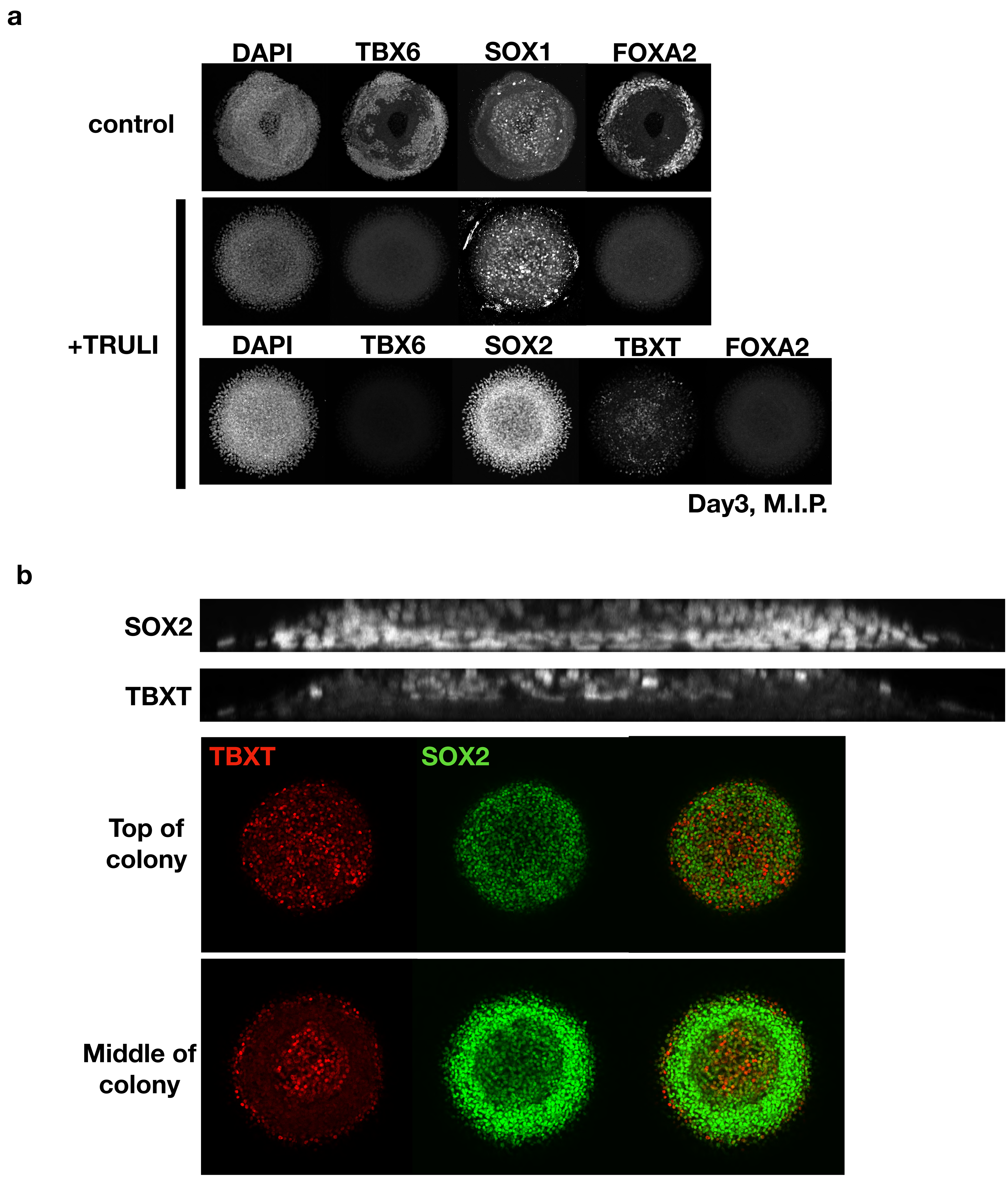

Supplementary Fig. 12

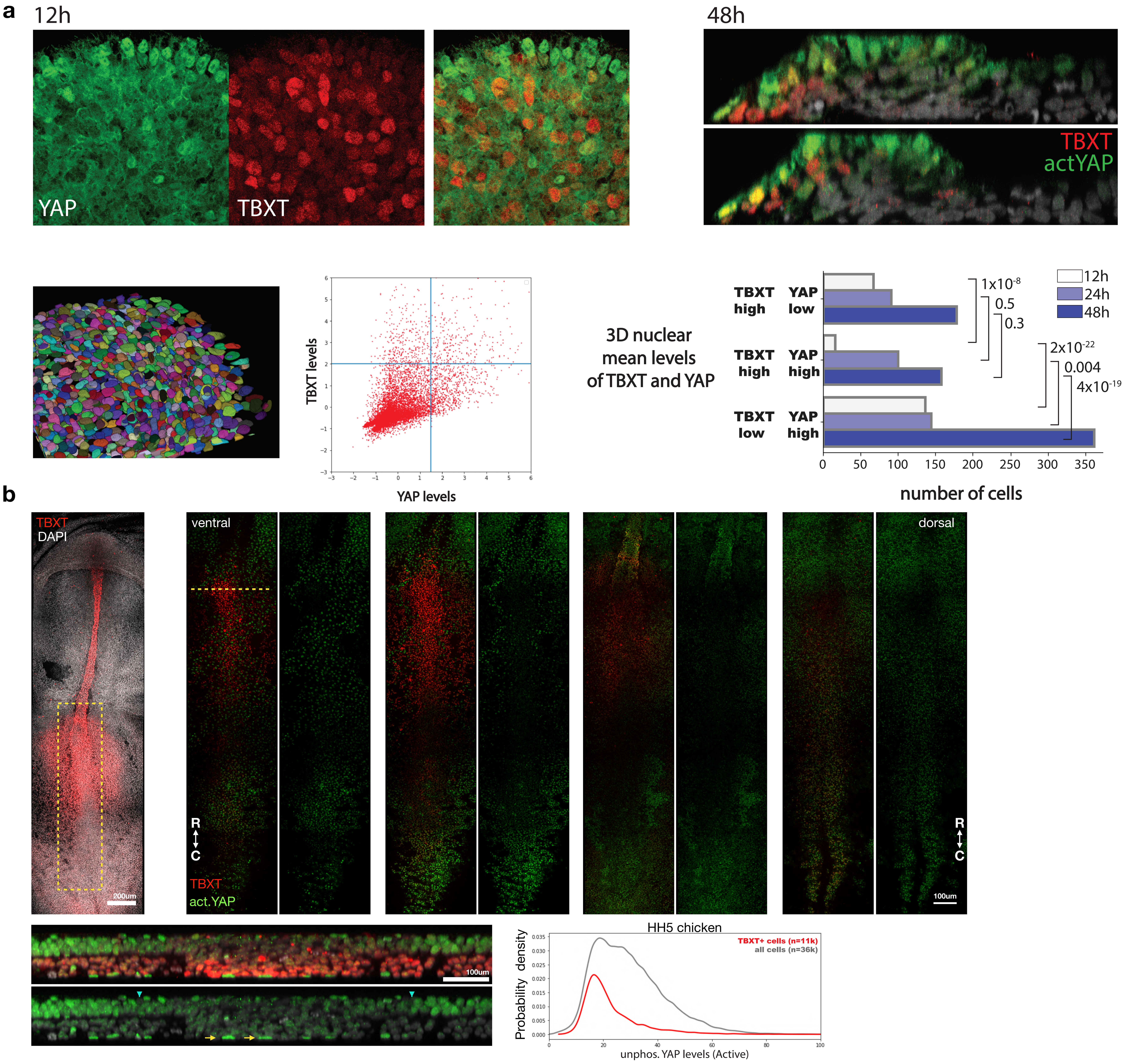

Supplementary Fig. 13

a

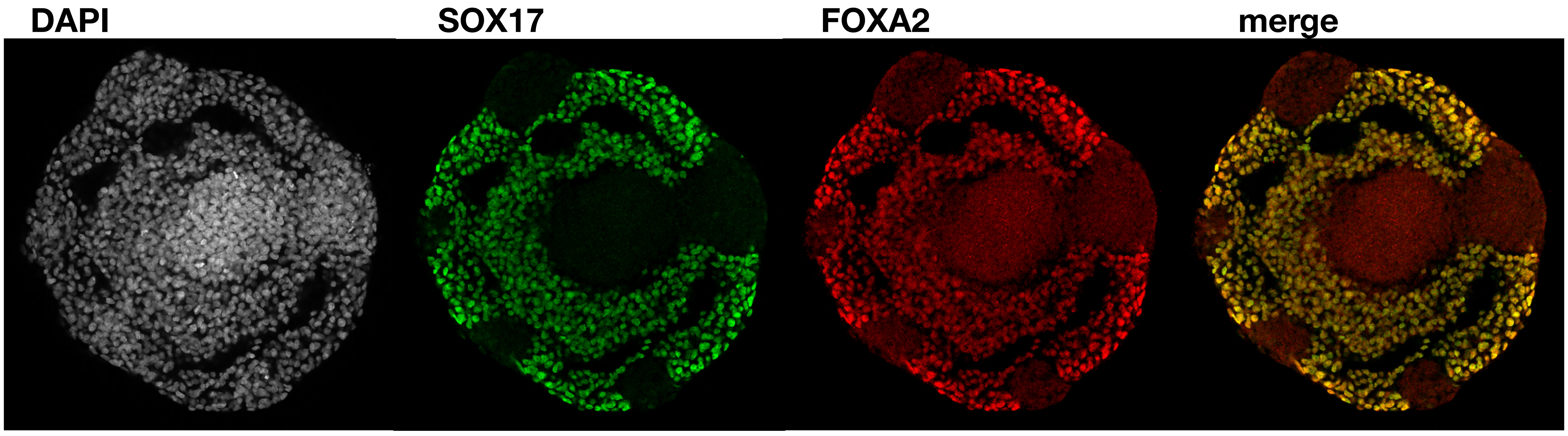

b

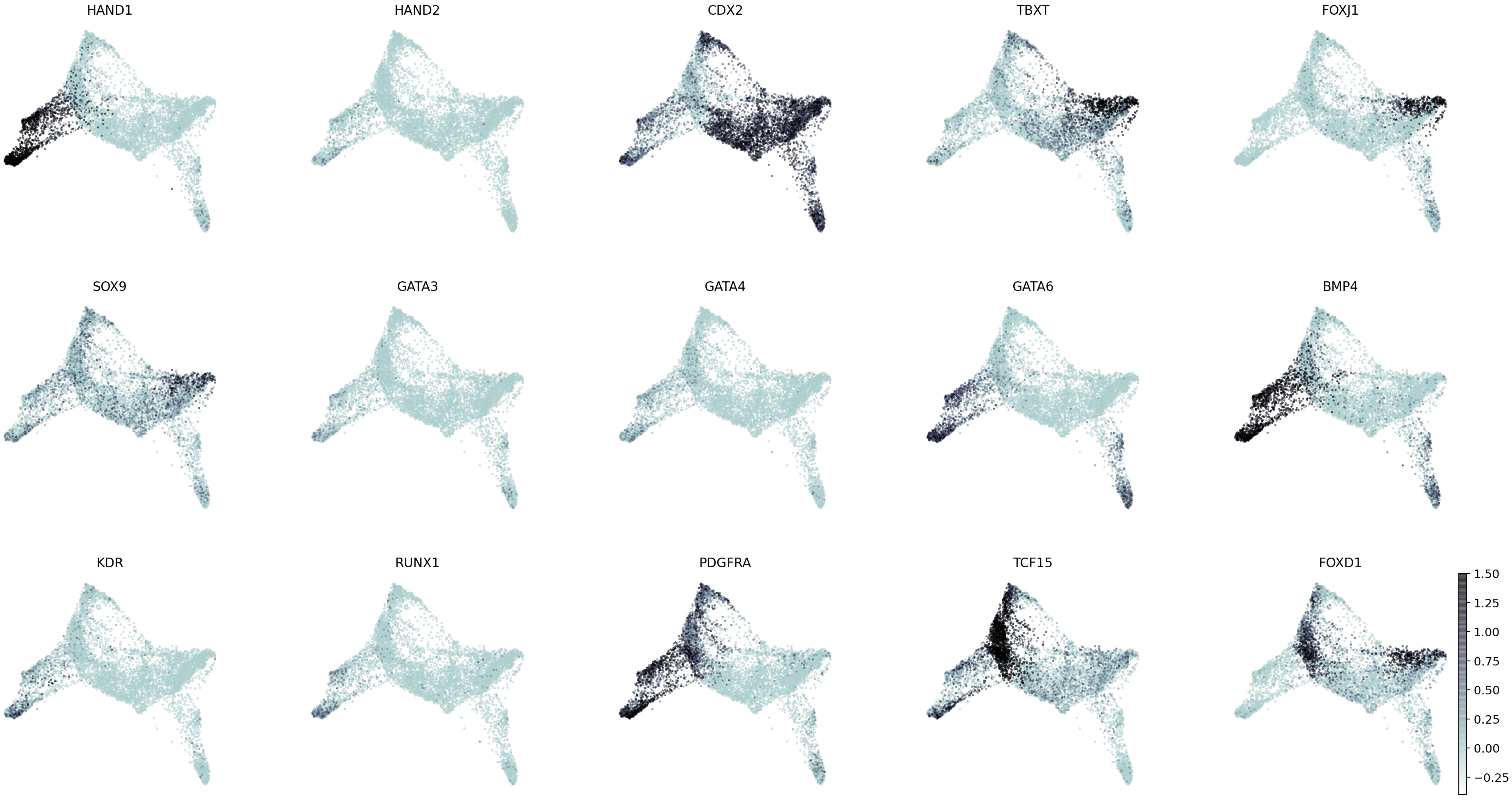

Supplementary Fig. 14

a

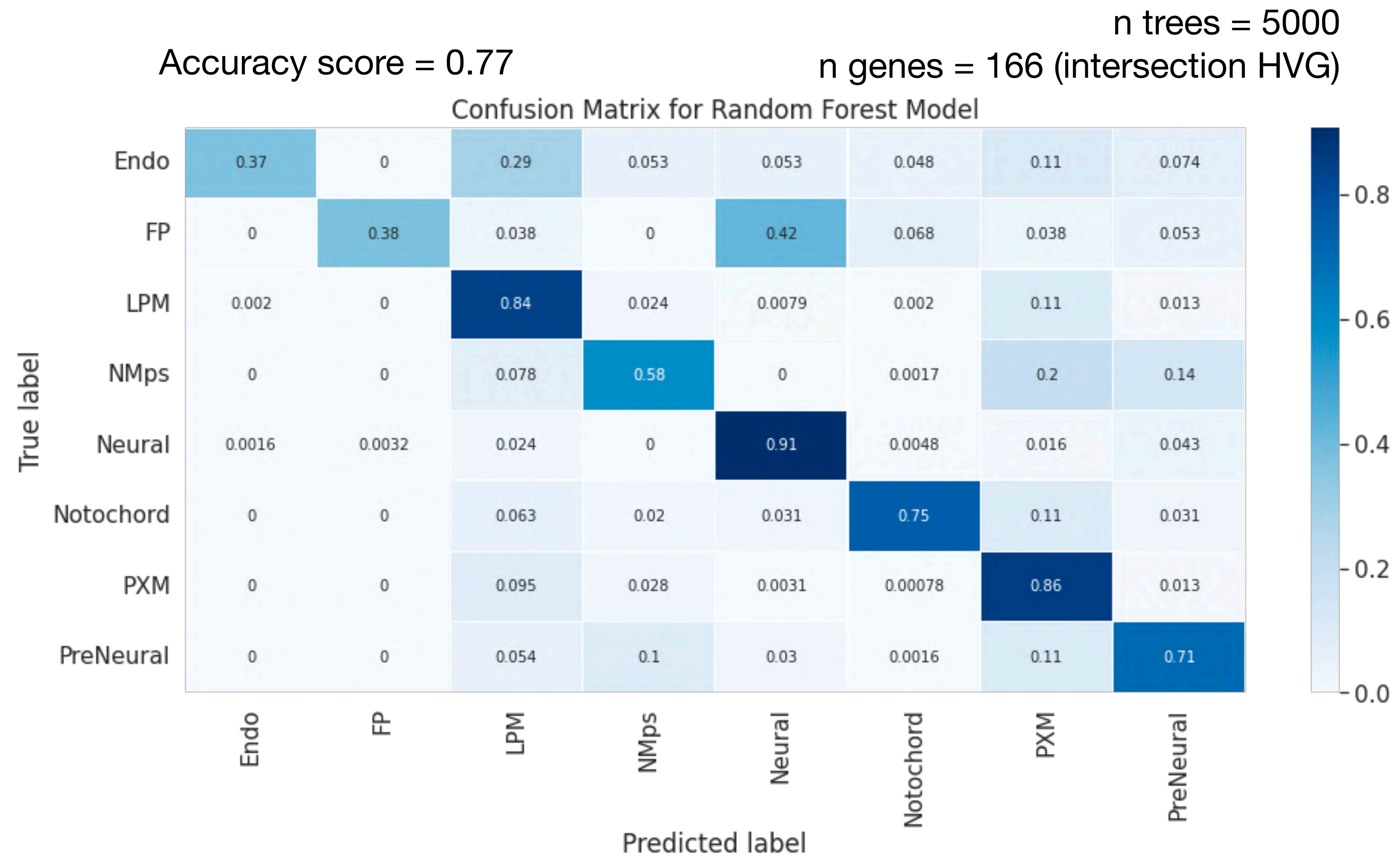

b

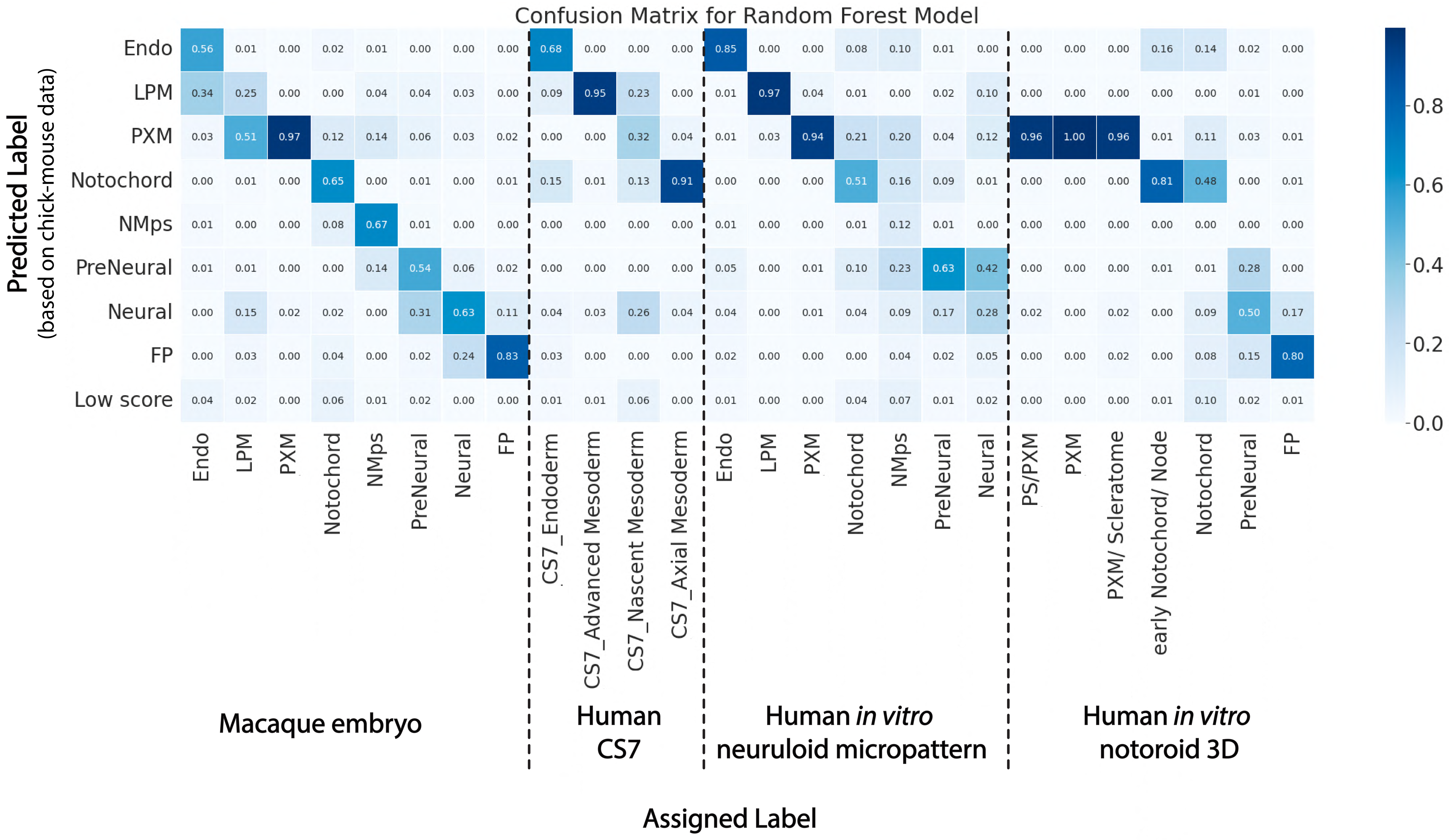

Supplementary Fig. 15

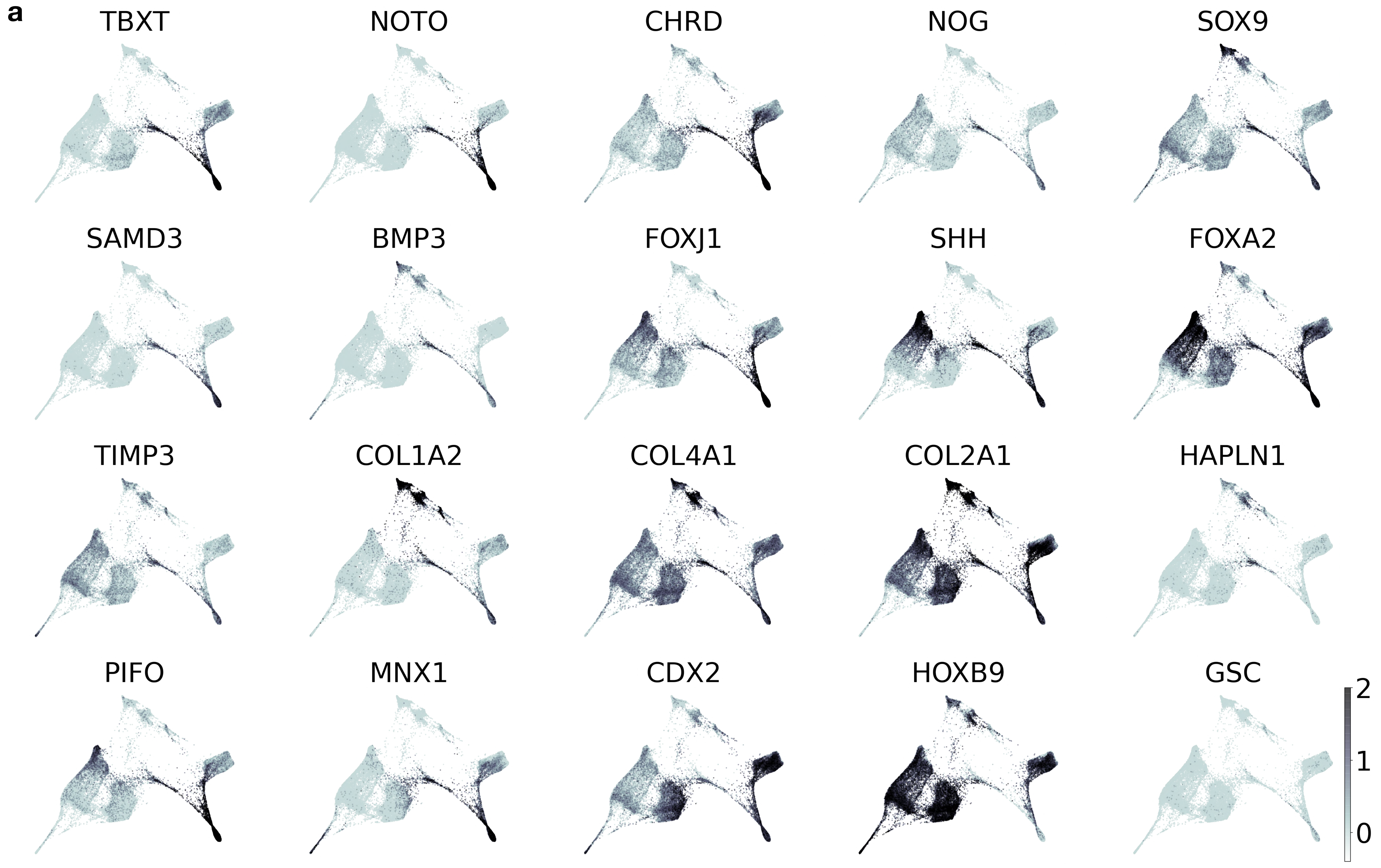

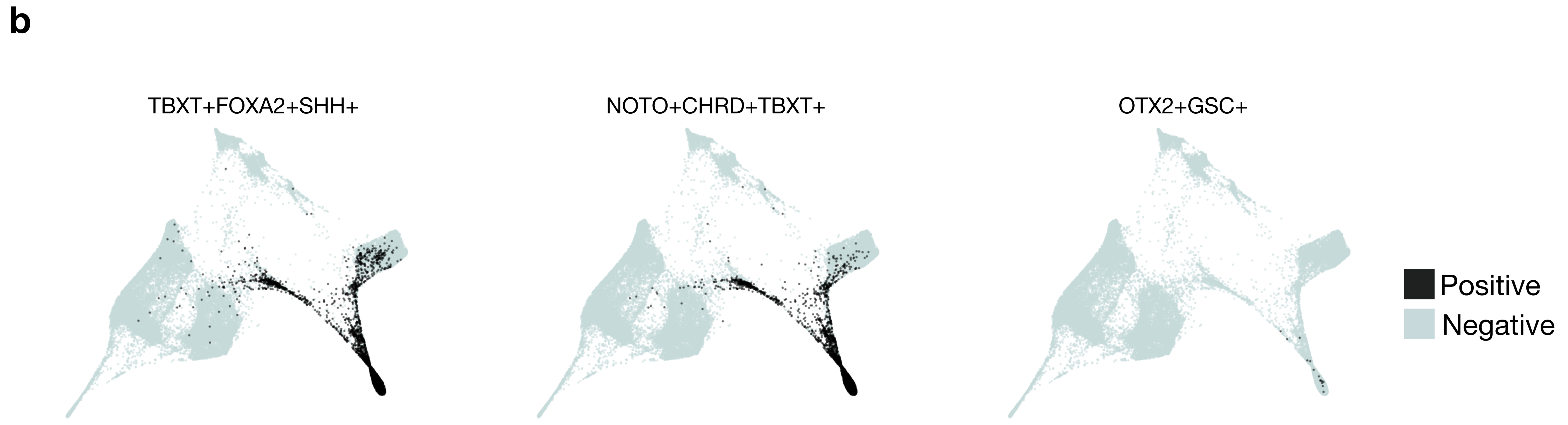

Supplementary Fig. 16

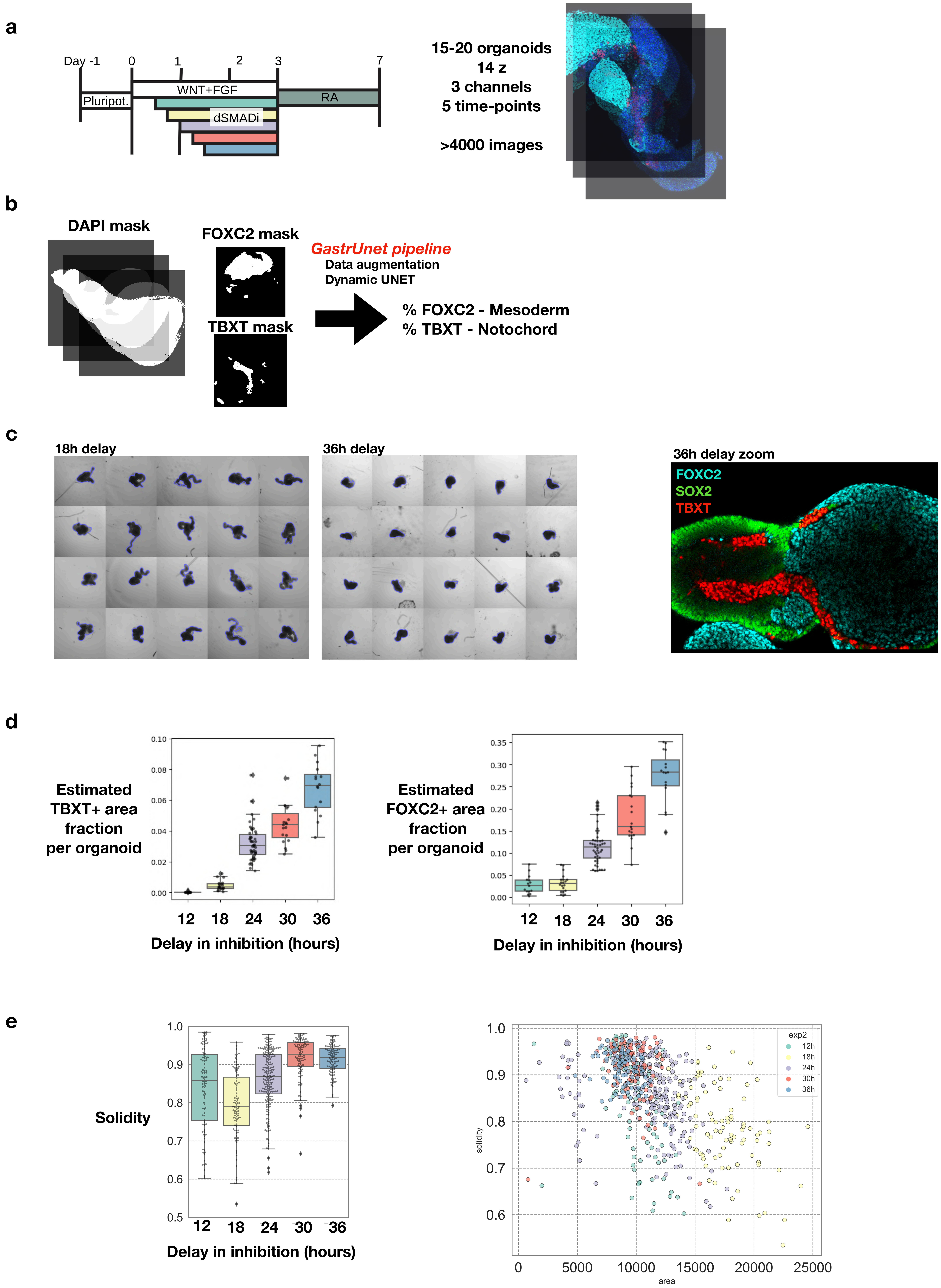

Supplementary Fig. 17

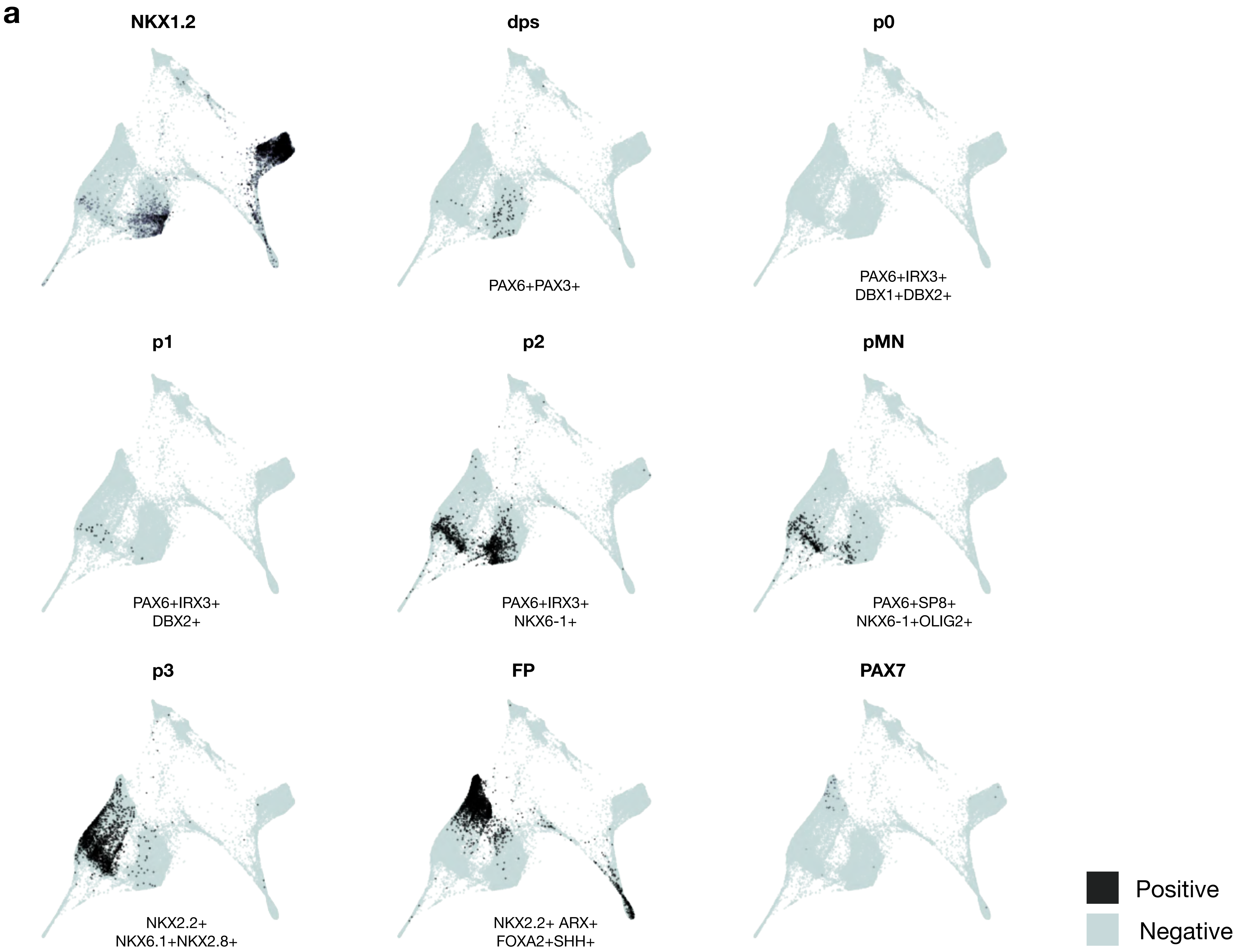

Supplementary Fig. 18

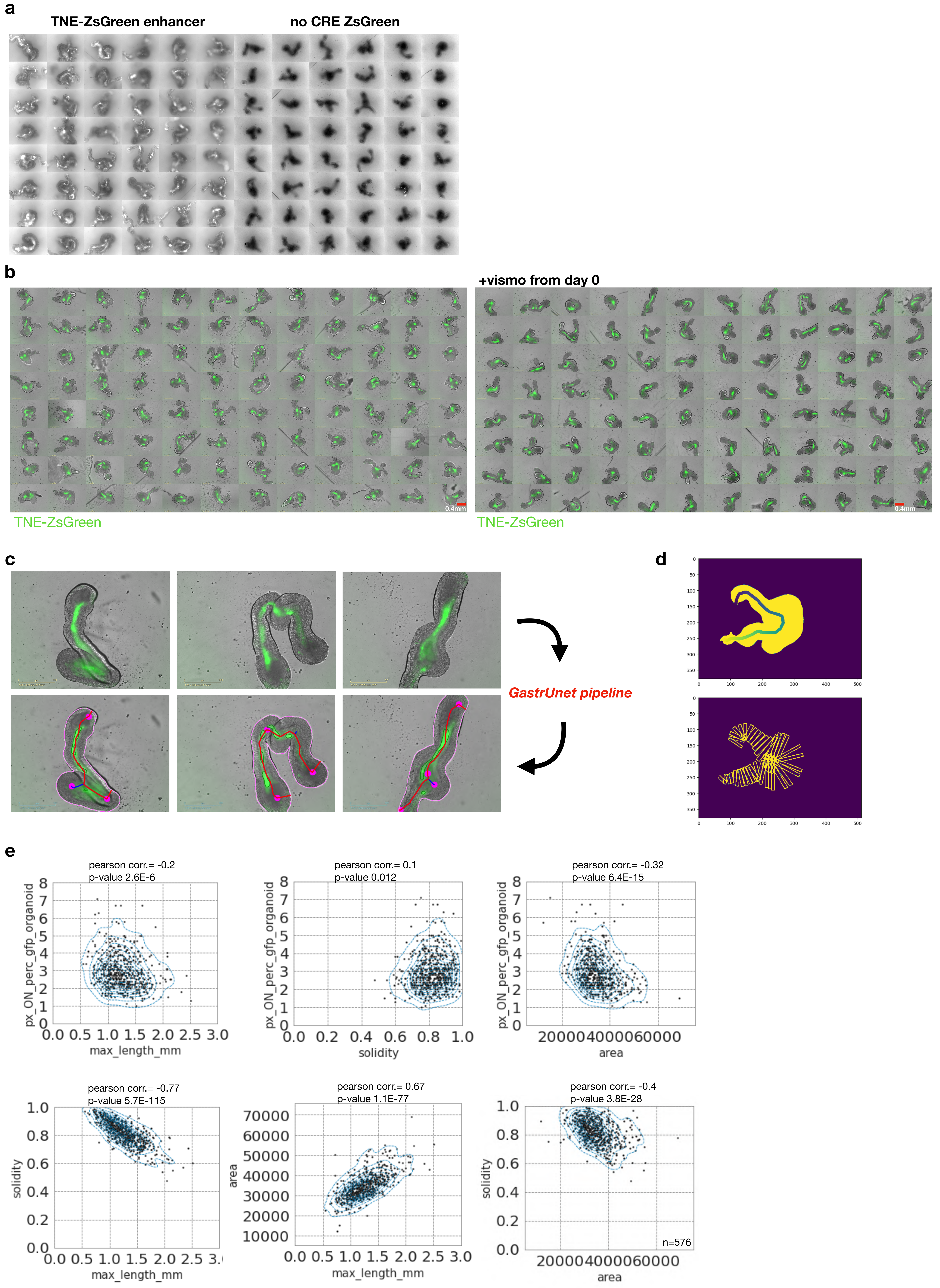

Supplementary Fig. 19

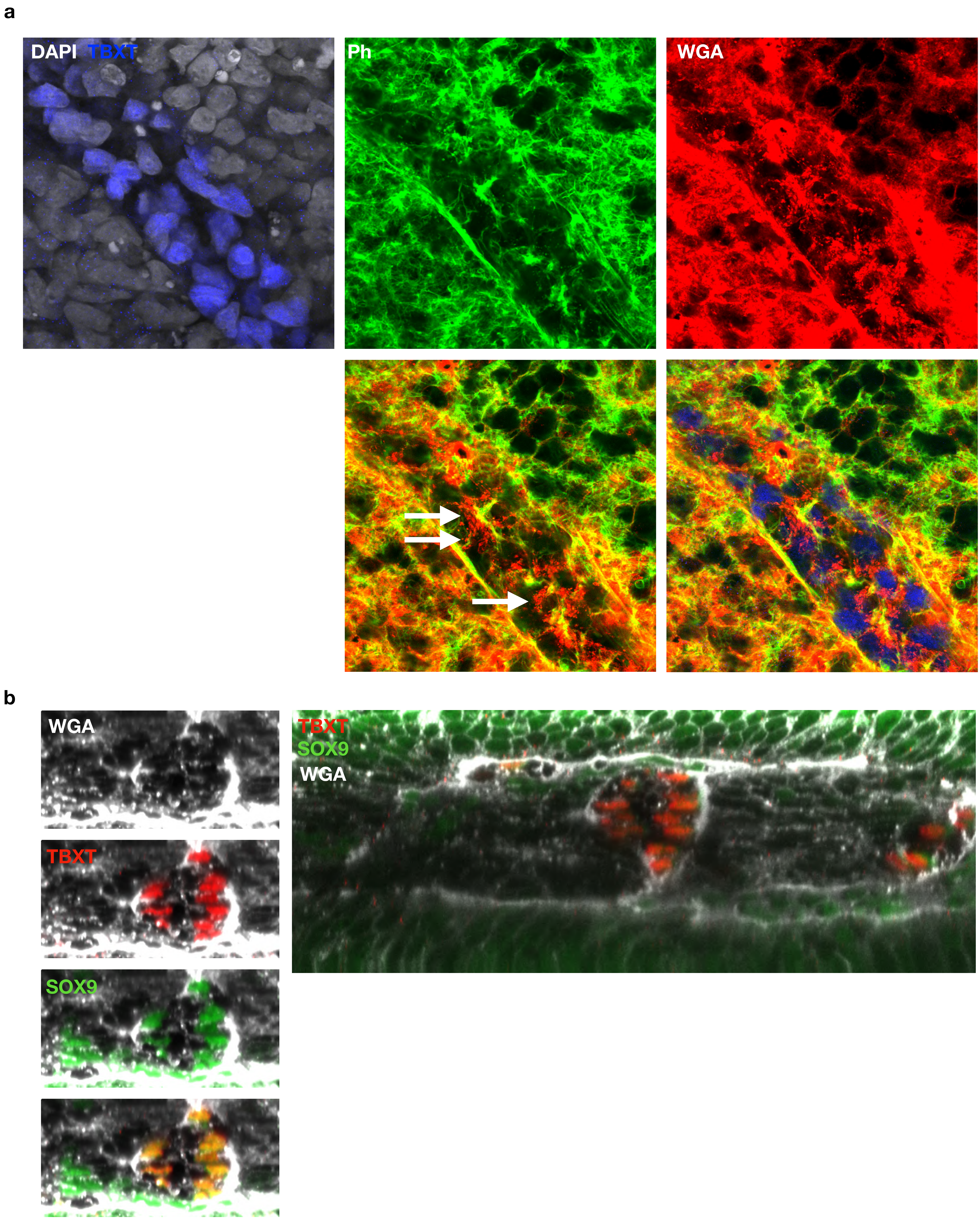

Supplementary Fig. 20

A. Features of notochord cells in notoroids

Desmosomes

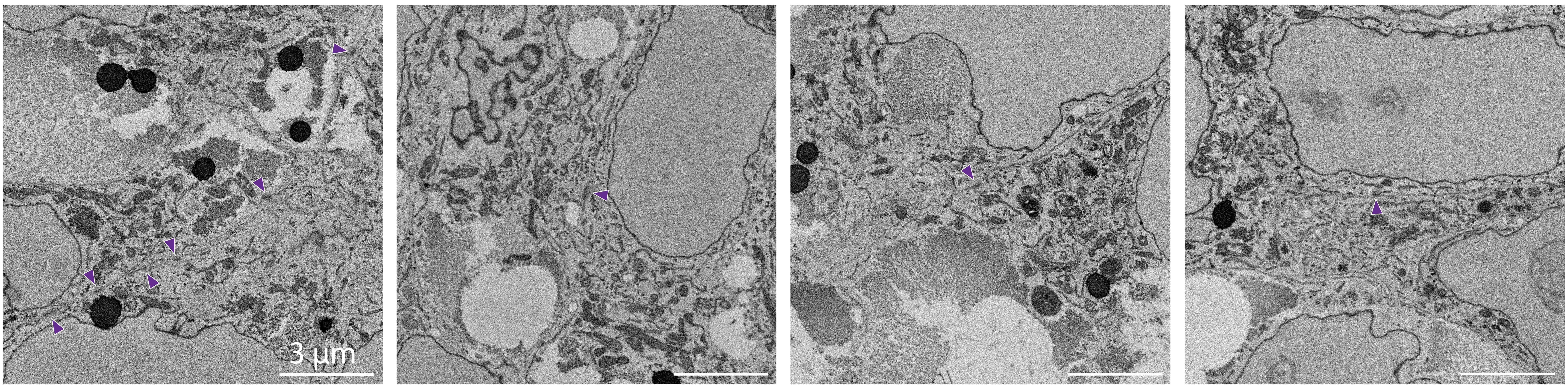

Cilia

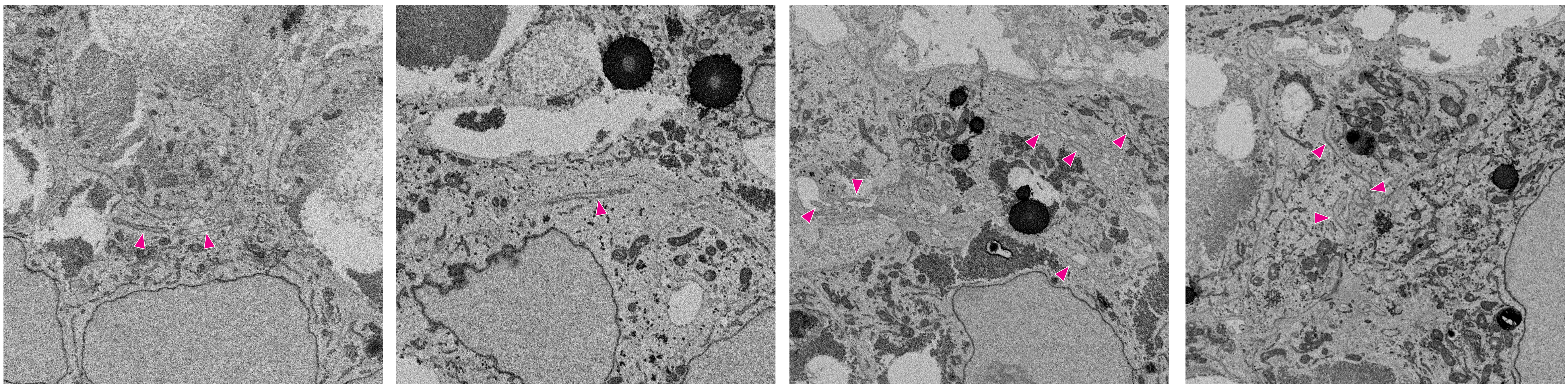

Dilated ER

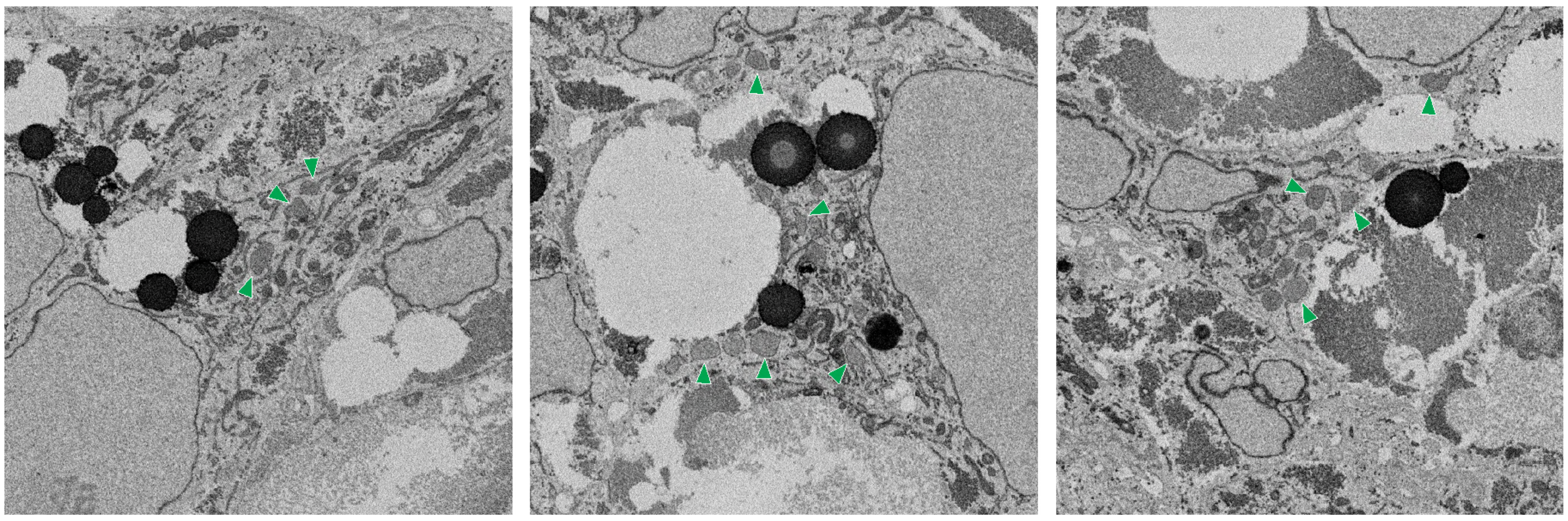

ECM fibers

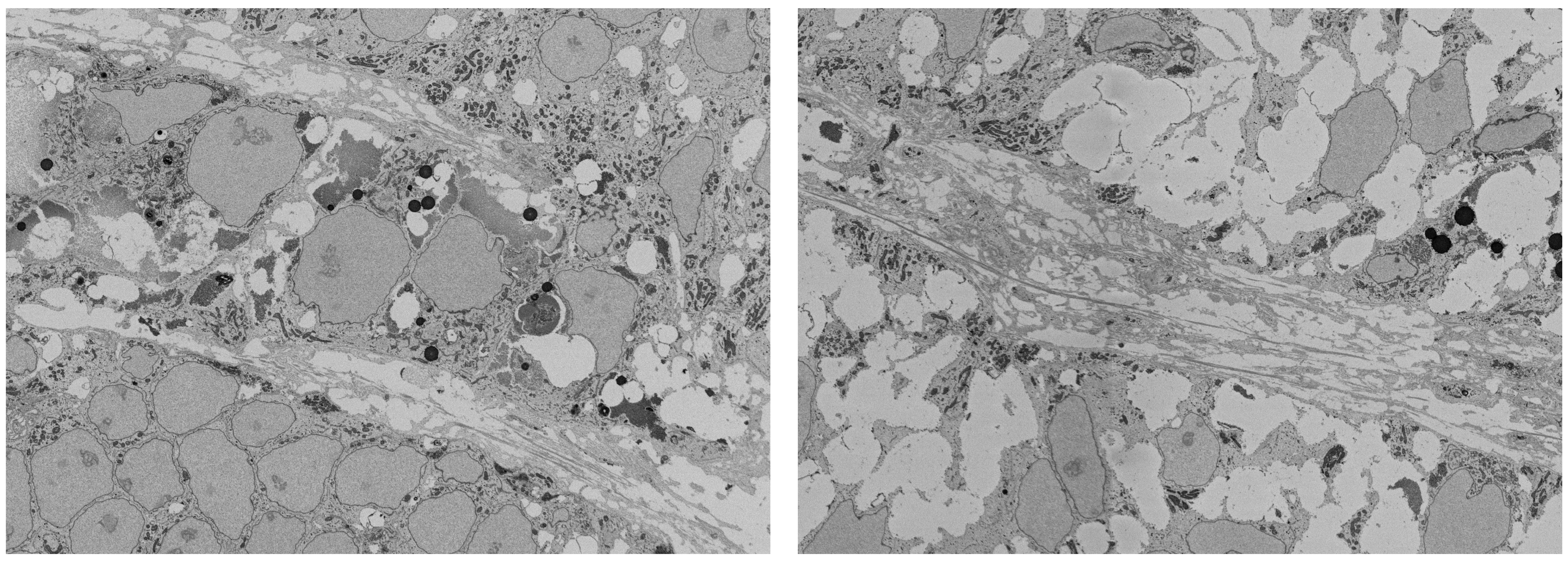

Vacuoles

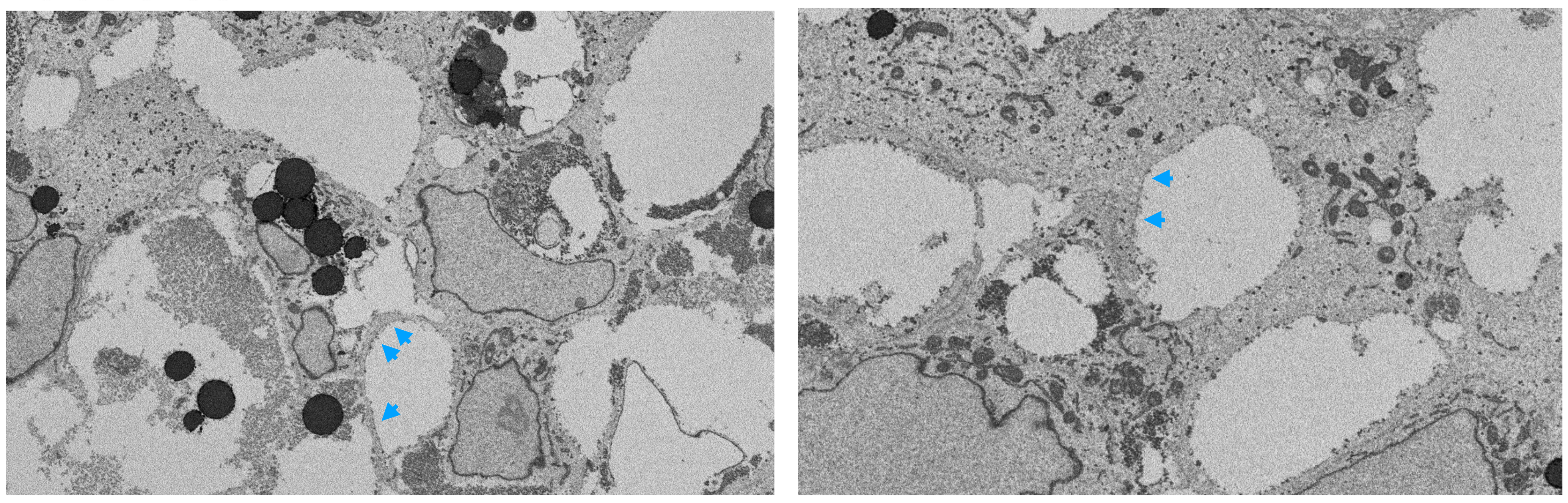

Supplementary Fig. 21

A. Neural cells

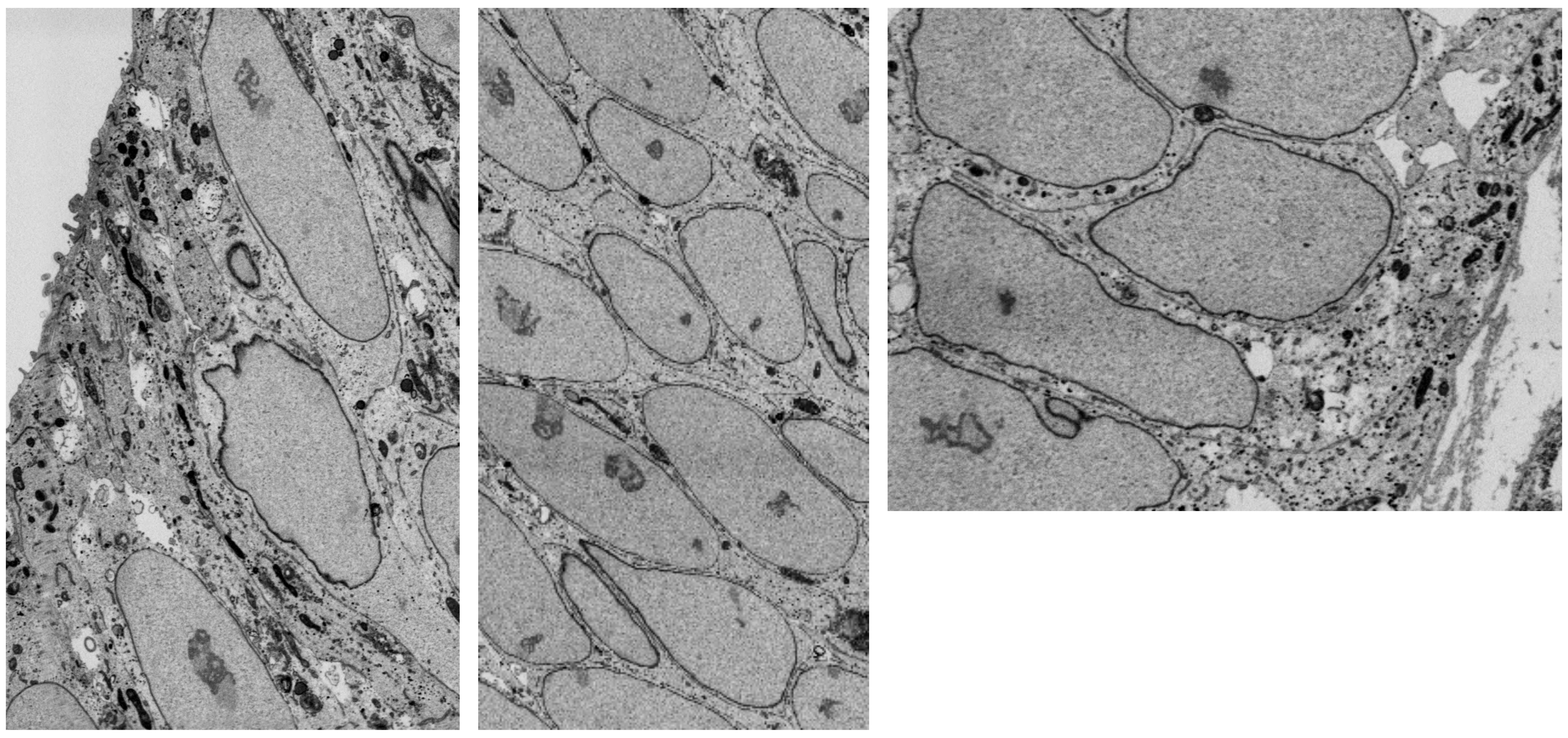

B. Mesenchymal cells

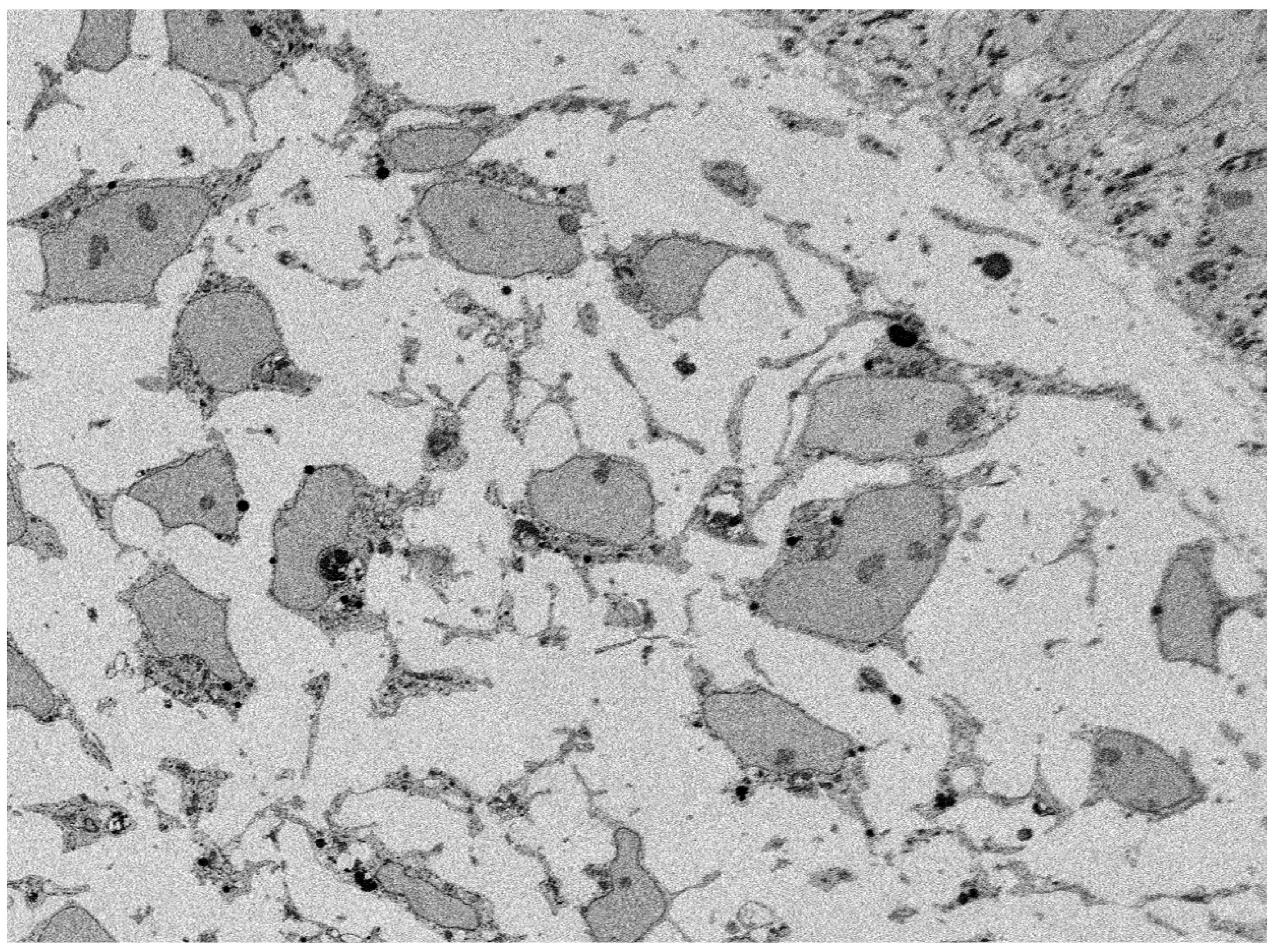

Supplementary Fig. 22

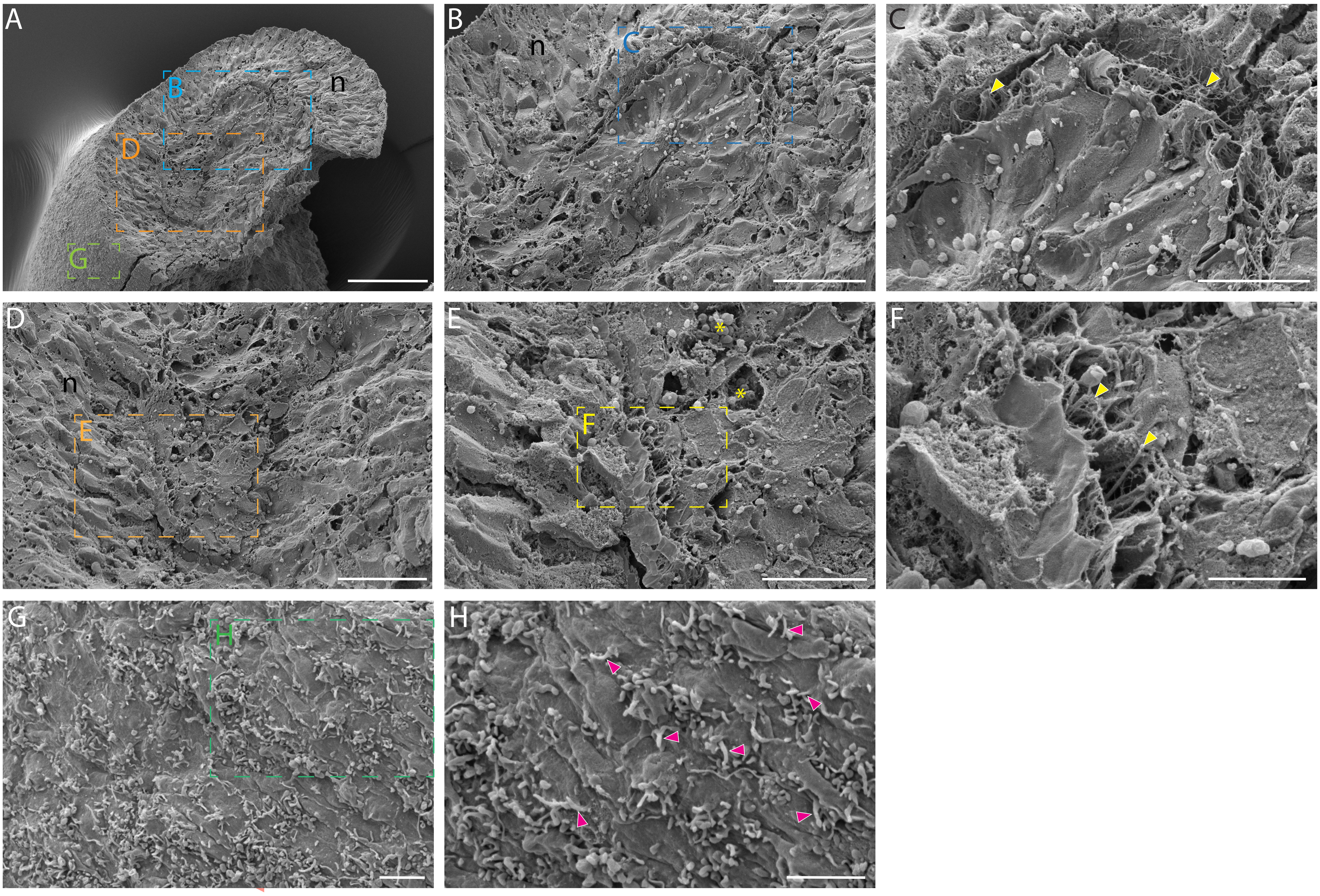

Supplement: Supplementary file 1 — Supplementary Figs 1–22. [file 41586_2024_8332_MOESM1_ESM.pdf]
